# A sodium/potassium switch for G4-prone G/C-rich sequences

**DOI:** 10.1093/nar/gkad1073

**Published:** 2023-11-20

**Authors:** Yu Luo, Martina Lenarčič Živković, Jiawei Wang, Jan Ryneš, Silvie Foldynová-Trantírková, Lukáš Trantírek, Daniela Verga, Jean-Louis Mergny

**Affiliations:** Laboratoire d’Optique et Biosciences, Ecole Polytechnique, CNRS, Inserm, Institut Polytechnique de Paris, 91128 Palaiseau, France; CNRS UMR9187, INSERM U1196, Université Paris-Saclay, F-91405 Orsay, France; Central European Institute of Technology, Masaryk University, 625 00 Brno, Czech Republic; Slovenian NMR Centre, National Institute of Chemistry, SI-1000 Ljubljana, Slovenia; Laboratoire d’Optique et Biosciences, Ecole Polytechnique, CNRS, Inserm, Institut Polytechnique de Paris, 91128 Palaiseau, France; Central European Institute of Technology, Masaryk University, 625 00 Brno, Czech Republic; Central European Institute of Technology, Masaryk University, 625 00 Brno, Czech Republic; Central European Institute of Technology, Masaryk University, 625 00 Brno, Czech Republic; CNRS UMR9187, INSERM U1196, Université Paris-Saclay, F-91405 Orsay, France; CNRS UMR9187, INSERM U1196, Institut Curie, PSL Research University, F-91405 Orsay, France; Laboratoire d’Optique et Biosciences, Ecole Polytechnique, CNRS, Inserm, Institut Polytechnique de Paris, 91128 Palaiseau, France

## Abstract

Metal ions are essential components for the survival of living organisms. For most species, intracellular and extracellular ionic conditions differ significantly. As G-quadruplexes (G4s) are ion-dependent structures, changes in the [Na^+^]/[K^+^] ratio may affect the folding of genomic G4s. More than 11000 putative G4 sequences in the human genome (hg19) contain at least two runs of three continuous cytosines, and these mixed G/C-rich sequences may form a quadruplex or a competing hairpin structure based on G-C base pairing. In this study, we examine how the [Na^+^]/[K^+^] ratio influences the structures of G/C-rich sequences. The natural G4 structure with a 9-nt long central loop, CEBwt, was chosen as a model sequence, and the loop bases were gradually replaced by cytosines. The series of CEB mutations revealed that the presence of cytosines in G4 loops does not prevent G4 folding or decrease G4 stability but increases the probability of forming a competing structure, either a hairpin or an intermolecular duplex. Slow conversion to the quadruplex *in vitro* (in a potassium-rich buffer) and cells was demonstrated by NMR. ‘Shape-shifting’ sequences may respond to [Na^+^]/[K^+^] changes with delayed kinetics.

## Graphical Abstract

**Graphical Abstract:**
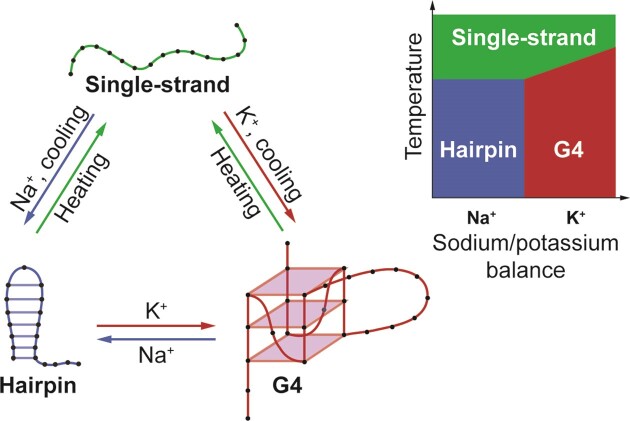

## Introduction

Metal ions are essential for sustaining plant, animal and human life. Metal ions are involved in intracellular and intercellular communication, the preservation of electrical charges and osmotic pressure, photosynthesis and electron transfer activities, and, for nucleic acids, the maintenance of base pairing and stacking (1). The most prevalent metal ions in the human body—as well as in most living species—are potassium and sodium. Sodium is one of the most crucial electrolytes in the extracellular fluid, while potassium is mainly an intracellular ion. The sodium-potassium ATP pumps (Na⁺/K⁺-ATPase), located in the plasma membrane of almost every cell, export three sodium ions in exchange for two potassium ions, and this active transport process is primarily responsible for controlling the balance between sodium and potassium (2). Potassium/sodium balance helps to maintain vital body functions. In contrast, potassium/sodium imbalance contributes to several diseases. For example, sodium intake has been tightly related to blood pressure and hypertension (3), while intracellular [Na^+^] and [K^+^] levels are both increased in brain regions of patients affected by Alzheimer disease (4).

G-quadruplexes (G4s) are four-stranded nucleic acid secondary structures constituted by two or more stacked G-quartets. G4s have been shown to be involved in DNA replication (5), gene transcription (6), and pre-mRNA alternative splicing (7). As topology and stability of G4 structures are both sensitive to the cation type and ionic strength (8), putative quadruplex sequences (PQS) in different potassium/sodium ratio environments may adopt distinct conformations, resulting in diverse effects on cell activities. During tumor progression, G4 structures may influence gene expression in response to changes in intracellular cation concentrations. Due to the overexpression of a [K^+^] channel, malignant cancer cells (*i.e*. highly metastatic breast cancer cells MDA-MB-231) may exhibit a significantly lower intracellular [K^+^] than normal cells. In normal cells, high [K^+^] has been proposed to inhibit the transcription of certain oncogenes by stabilizing G4 structures, whereas in cancer cells, lower [K^+^] may destabilize G4s and increase the transcription of oncogenes (9). This result is surprising, given that cancer cells seem to exhibit more G4s, as shown by BG4 antibody immunostaining experiments (10). A recent study reported that potassium/sodium balance is important to regulate alternative promoter usage and/or pre-mRNA splicing in the transcription of SGK1, via folding a G4 structure located in its promoter. The SGK1 gene has a G4-forming motif located upstream to the proximal region of promoter-2, which has been shown to be stabilized by potassium ions and resveratrol but destabilized by sodium ions. For all three SGK1 isoforms, transcription is stimulated by high sodium levels, whereas resveratrol or potassium ion addition suppresses the transcription of isoform-2 and isoform-3, but not isoform-1 (11). G4-Hairpin equilibrium may also be affected by the presence of G4-ligands (12).

Characterizing the structures formed by G-rich strands in multi-ion environments may help understand how G4 folding influences gene activities. However, G4 formation is often studied under simple monocationic conditions, which strongly differ from the real intracellular conditions. In addition, a number of potential quadruplex sequences contain runs of cytosines directly upstream or downstream of the G-rich sequence or in the loops. For example, we found that about 10% of PQSs in the human genome (hg19) involve at least three continuous cytosines. These mixed G/C-rich sequences may form a quadruplex or a competing hairpin structure based on G-C base pairing, and this competition may be relevant for RNA sequences as well (13,14). The relative stabilities of these competing structures should depend on the sequence but also on ionic conditions, given that G4 stability should be dependent on potassium/sodium balance while the formation of hairpin should not.

**Scheme 1.**
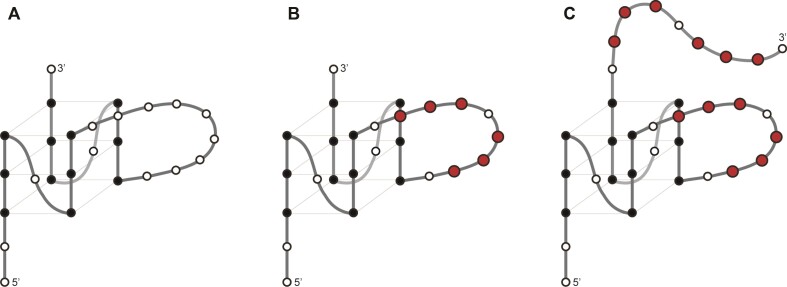
Possible conformations of the long-loop G4s. Guanines in the G-quartet cores, loop residues and cytosines are colored black, white and red, respectively. (**A**) G4 topology adopted by the wt sequence: CEBwt occupies a three-quartet parallel G4 topology with two 1-nt loops and a 9-nt central loop. (**B**) Positions in which nucleotides are replaced by cytosines in CEBm4 are indicated in red. (**C**) Hypothetical G4 conformation that may be adopted by CEBm7. Cytosines present on the 3′- extension are shown in red. A high resolution structure for CEBwt is available (PDB 2LPW).

In this study, we worked on variants of the well-known human minisatellite G4 structure CEBwt in which we progressively inserted runs of consecutive cytosines. The structure of the CEBwt quadruplex was previously solved by NMR (PDB: 2LPW): it corresponds to a parallel intramolecular DNA G4 with a 9-nt long central loop (15); the possible conformations of three long-loop G4s are shown in Scheme 1. We analyzed G4 and hairpin formation under different potassium/sodium ion ratios for CEBwt and its mutants. Starting from this sequence, we introduced base substitutions to increase the share of cytosines in CEBwt variants to identify the factors affecting the equilibrium between G4 and hairpin formation under complex ionic conditions. We identified ‘shape-shifting’ sequences that respond to K^+^/Na^+^ balance and are expected to adopt different folds in the intra- and inter-cellular environment.

## Materials and methods

### Materials and samples

Most oligonucleotides were purchased from Eurogentec (Belgium) and used without further purification; their sequences are shown in Table 1. Stock solutions were prepared at 100 μM strand concentration in milli-Q H_2_O. All oligonucleotides were annealed in corresponding buffers, kept at 95°C for 5 min and slowly cooled to room temperature before measurements. All chemical reagents, including Hemin, ABTS^2−^ and H_2_O_2_, were purchased from Sigma-Aldrich (France). Deuterated cacodylic acid (d7, 98%) used to prepare the lithium cacodylate buffer for NMR spectroscopy was purchased from Cambridge Isotope Laboratories, Inc. (USA).

**Table 1.** Oligonucleotide sequences derived from CEBwt

Acronym	Sequence (5′- 3′) ^a^	Length (nt)	G4H score^b^	*N* ^c^
CEBwt	AAGGGTGGGTGTAAGTGTGGGTGGGT	26	1.50	0
CEBm0A	AAGGGTGGGTAAAAAAATGGGTGGGT	26	1.38	0
CEBm0T	AAGGGTGGGTTTTATTTTGGGTGGGT	26	1.38	0
CEBm1	AAGGGTGGGT**CCC**AGTGTGGGTGGGT	26	1.12	1
CEBm2	AAGGGTGGGT**CCC**A**C**TGTGGGTGGGT	26	1.04	1.33
CEBm3	AAGGGTGGGT**CCC**A**CC**GTGGGTGGGT	26	0.92	1.67
CEBm4	AAGGGTGGGT**CCC**A**CCC**TGGGTGGGT	26	0.69	2
CEBm5	AAGGGTGGGT**CCC**A**CCC**TGGGTGGGT**CCC**A	30	0.30	3
CEBm6	AAGGGTGGGT**CCC**AGTGTGGGTGGGT**CCC**A**CCC**A	34	0.32	3
CEBm7	AAGGGTGGGT**CCC**A**CCC**TGGGTGGGT**CCC**A**CCC**A	34	0	4

^a^The cytosines introduced in the mutant sequences are marked in bold characters. ^b^ G4Hunter score, calculated according to Bedrat *et al.* (23). ^c^ ‘*N*’ represents numbers of three-continuous-cytosines (‘CCC’) motifs.

The CEBm4 oligonucleotide used for NMR experiments was synthesized on a DNA/RNA H-8 Synthesizer (K&A Laborgeräte GbR) using standard phosphoramidite chemistry with DMT protecting group. The oligonucleotide was cleaved from the solid support and deprotected with ammonium hydroxide and methylamine in a 1:1 (v/v) ratio for 30 min at room temperature and 30 min at 65°C. Samples were purified using GlenPak cartridges (Glen Research) and desalted on FPLC with HiPrep 26/10 Desalting column (GE Healthcare). Samples were dried using a vacuum centrifuge and afterward dissolved in deionized water. The concentration of the stock solution was determined by UV absorption at 260 nm using a Varian Cary 100 Bio UV/VIS spectrophotometer.

### Size-exclusion high-performance liquid chromatography (SE-HPLC)

SE-HPLC experiments were performed on a ÄKTA FPLC 900 system (GE Healthcare, France) equipped with a Frac-900 autosampler, a Thermo Acclaim SEC-300 column (4.6 × 300 mm; 5 μm hydrophilic polymethacrylate resin spherical particles with 300 Å pore size), and a diode array detector. A solution containing 40 mM cacodylate buffer, pH 6, with 100 mM potassium was used as an elution buffer and to dissolve oligonucleotides. 20 μl of a 50 μM oligonucleotide solution in 50 mM Tris·HCl (pH 7.2) with 140 mM NaCl or 140 mM KCl was injected onto the column (0.15 ml/min elution flow rate at 20°C), and elution was monitored by measuring the absorbance at 260 nm.

### UV-melting

Oligonucleotides (5 μM strand concentration) were pre-annealed in 10 mM lithium cacodylate (LiCaco) buffer (pH = 7.2) complemented with different concentrations of potassium/sodium ions. We defined nine different buffer conditions, from B1 to B9, as follows:

B1: 140 mM Na^+^/ 0 mM K^+^; B2: 135 mM Na^+^/ 5 mM K^+^; B3: 125 mM Na^+^/ 15 mM K^+^; B4: 115 mM Na^+^/ 25 mM K^+^; B5: 100 mM Na^+^/ 40 mM K^+^; B6: 80 mM Na^+^/ 60 mM K^+^; B7: 70 mM Na^+^/ 70 mM K^+^; B8: 20 mM Na^+^/ 120 mM K^+^; B9: 0 mM Na^+^/ 140 mM K^+^.

UV-melting curves were recorded with a Cary 300 (Agilent Technologies, France) spectrophotometer. Heating runs were performed between 10°C and 95°C, the temperature was increased by 0.2°C per minute, and absorbance was recorded at 260 and 295 nm. *T_m_* was determined as the temperature corresponding to half of the height of the melting curve.

### Thermal difference spectra (TDS)

Absorbance spectra of pre-folded CEBm2 in B3 and CEBm3 in B4 buffer were recorded on a Cary 300 (Agilent Technologies, France) spectrophotometer at 25°C and 95°C (scan range: 400–200 nm; scan rate: 600 nm/min; automatic baseline correction). Oligonucleotide concentration was set to 5 μM in a final volume of 1 ml. TDS corresponds to the arithmetic difference between the initial (25°C) and second (95°C) spectra.

### Circular dichroism (CD) measurements

CD spectra of pre-folded 5 μM oligonucleotides in 1 ml of different buffers were recorded on a JASCO J-1500 (France) spectropolarimeter (scan range: 340–200 nm; scan rate: 100 nm/min; averaging three accumulations) at 25°C or 37°C for isothermal experiments.

### 1D ^1^H NMR analysis

NMR spectra of CEBm4 were recorded at 25 or 37°C on a 600 MHz Bruker Avance NEO NMR spectrometer equipped with a quadruple resonance cryoprobe. Samples of CEBm4 used to assess the influence of ion composition on folding were dissolved at 0.2 mM concentration in a 10 mM lithium cacodylate (d7) buffer (pH 7.2) in 90% H_2_O/10% ^2^H_2_O complemented with different concentrations of sodium/potassium ions, namely B1, B2, B5, B7, and B9 (*vide supra*). Samples used for time-resolved NMR experiments were either dissolved in B1 (10 mM lithium cacodylate (d7), pH 7.2, 140 mM NaCl; marked as w/NaCl) or in 10 mM lithium cacodylate (d7), pH 7.2 without NaCl (referred to as w/o NaCl) at 0.2 mM DNA concentrations. All samples were then heated to 95°C for 5 minutes and gradually cooled to room temperature overnight. We then diluted the latter two samples to 0.1 mM DNA concentration (and 70 mM NaCl in the case of w/NaCl), added 70 mM KCl without further annealing and monitored G4 formation at different time points: 5, 10, 15, 20, 30, 40, 60 min, 2, 3, 4, 5, 6, 9, 12, 15, 18, 24 h and 3 days after KCl addition at 37°C. During that time, both samples were kept at 37°C either inside the spectrometer, or in a water bath.

### In-cell NMR spectroscopy

A sample for in-cell NMR measurement was prepared as described in (16). Briefly, HeLa cells were electroporated with 400 μM non-labelled DNA oligonucleotide (d-AAGGGTGGGTCCCACCCTGGGTGGGT) supplemented with 10 μM 5′-FAM labelled oligonucleotide in electroporation buffer (140 mM Na_2_HPO_4_/NaH_2_PO_4_, 5 mM KCl, 10 mM MgCl_2_, pH 7.0), using electric pulses 1000 V, 100 μs followed by 350 V, 30 ms (BTX-ECM 830 system; Harvard Apparatus, USA). After electroporation, FAM intensity in cells was monitored with flow cytometry (BD FACS Verse Cell Analyzer; BD Biosciences, USA) to check transfection efficiency and propidium iodide staining was used to determine cell survival. Subcellular localization of the introduced oligonucleotide and cell morphology was monitored using confocal microscopy (LSM 800; Carl Zeiss, Germany). For in-cell NMR, the cells were suspended in Leibovitz L15–/– medium containing 10% D_2_O, transferred into a 5 mm NMR cuvette, and pelleted by manual centrifugation to concentrate in active coil volume. 1D ^1^H in-cell NMR spectra were acquired at Bruker Avance NEO 950 MHz NMR (Bruker Corporation, Billerica, MA, USA) using a 1D ^1^H JR-echo (1–1 echo) pulse sequence (17) with zero excitation set to the resonance of water and the excitation maximum set to 12.5 ppm.

### DNAzyme activity assay

Hemin was dissolved in DMSO to obtain a solution at 1 mM concentration and stored in the dark at –20°C. ABTS^2−^and H_2_O_2_were freshly prepared in water at the desired concentration for use. DNA samples were folded by heating at 95°C for 5 min in the corresponding buffer and left to cool at room temperature for at least two hours. 5 μl of a fresh dilution of Hemin at 60 μM (10% DMSO) were then added to reach a final Hemin concentration of 6 μM and the solutions were left to stand at room temperature (25°C) for 30 min. 5 μl ABTS^2−^ at 5 mM was then added to each sample to a final concentration of 500 μM, and basal absorbance was measured. To initiate the oxidation reaction, H_2_O_2_ was added to a final concentration of 50 μM, followed by quick mixing. 15 min later, the absorbance at 420 nm of the oxidized product ABTS^•−^ was monitored by a TECAN M1000 pro plate reader (France). The final sample contained 3 μM DNA, 6 μM Hemin, 1% DMSO, 500 μM ABTS^2−^ and 50 μM H_2_O_2_ in a total volume of 50 μl.

### *N*-Methyl mesoporphyrin IX (NMM) light-up probe

In each microwell containing 95 μl of 3.15 μM pre-folded oligonucleotides solubilized in corresponding buffers, 5 μl of NMM at 40 μM were added to reach a final concentration of 2 μM. The microplate was shaken for 1 min, and fluorescence was read immediately at 25°C. Fluorescence intensity was collected at 610 nm after excitation at 380 nm in a TECAN M1000 pro plate reader (France).

## Results

### Analysis of PQS containing runs of consecutive cytosines

A G-quadruplex (G4) is a four-stranded structure constituted by two or more G-tetrads. In this study, we only consider classical G4 structures potentially involving three tetrads, in line with the original G4-predicting algorithms (18), with the basic requirement of 4 runs of 3 or more continuous guanines. Two-tetrads quadruplexes are often of low thermal stability and will not be considered here (19).

In this study, we assumed that the presence of at least one run of ‘CCC’ motif (*N* ≥ 1) may allow the formation of three consecutive G-C bps with a neighboring G4 track, possibly interfering with G4 folding. To search for these motifs, we did not use G4Hunter, as it tends to exclude sequences having blocs of consecutive Cs (20–22), which contribute negatively to the G4Hunter score (23). As a consequence, the majority of PQS containing C-runs are disfavored by G4Hunter (for example, see the scores listed in Table 1). We, therefore, used the GSE133379 database, which is a comprehensive listing of PQS motifs in the human genome (hg19) (24), and includes four validated types of G4s: (i) the most classical G4s: three or more G-tetrads with short loops (1–7 nt) (18); (ii) G4s with long loops (8–15 nt) (25); (iii) G-stems with bulges (26) and (iv) Guanine-vacancy-bearing G4s (27). Compared to the first human PQS dataset reported in 2005 (18), many more PQS (1 506 353) have been included in GSE133379 (Table S1), and the average PQS frequency is 0.48 per 1000 bp in the whole genome. It is worth noting that about 10% of these PQS contain at least one run of three consecutive cytosines, and more than half are located in functional regions of the genome. Particularly, 44.2% of ‘CCC’-containing (*N* ≥ 1) PQS are located in introns and exons, implying the significance of studying the influence of C-rich segments on structures these PQS form (Figure S1). Then, we further increased the stringency of our PQS search to *N* ≥ 2, and found over 11000 PQS that meet this requirement (Table S1).

### Secondary structures of G/C-rich sequences in different Na^+^/K^+^ buffers

Having established that a number of PQS motifs containing one or more runs of cytosines are present in the genome, we wanted to investigate how they could affect G4 formation. Several cytosine-containing G4 structures have already been characterized (*e.g. VEGF* d[CG_4_CG_3_CCTTG_3_CG_4_T], PDB: 2M27 (28), implying that the presence of a few cytosines (*e.g*. one CC track) does not affect G4 formation and stability. We therefore considered motifs having longer runs (3 or more) of cytosines in the loops. To do so, we started with the well-known human minisatellite CEB25 (called CEBwt here) G4 structure involving a long (9 nucleotides) central loop (PDB: 2LPW (15), allowing us to introduce multiple bases substitutions in this region. With this in mind, we mutated the sequence by replacing loop residues with cytosines; the first mutation contains one run of the ‘CCC’ motif. SE-HPLC was first used to confirm the molecularity of CEBwt and each mutation. Single peaks corresponding to a retention time of around 18 min were recorded, in agreement with the formation of intramolecular structures in potassium and sodium buffers (Figure S2). CD spectra and UV-melting profiles were used to determine structure formation and *T_m_* values of CEBwt and its mutated variants.

**Figure 1.**
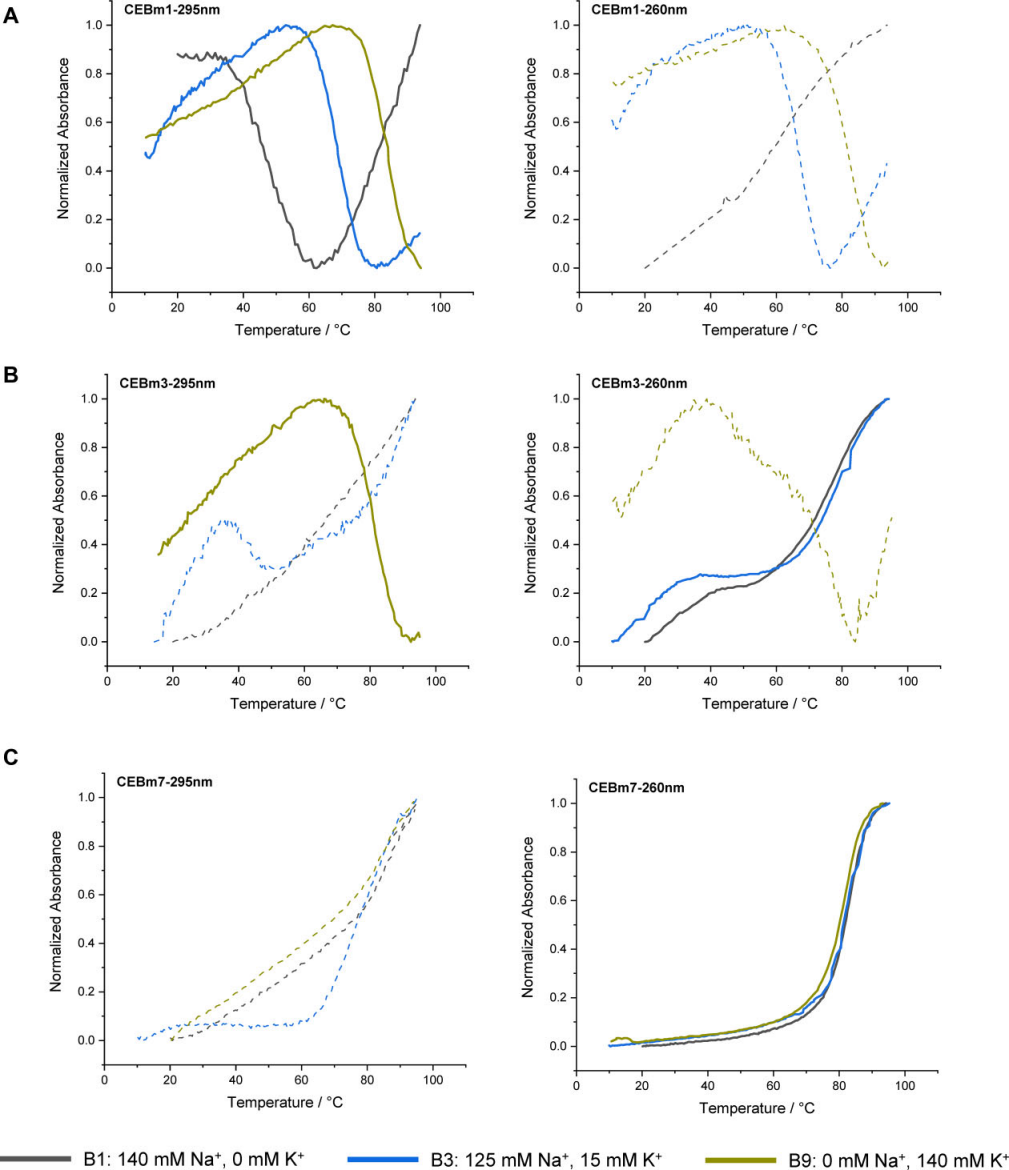
Normalized UV-melting curves of 5 μM (**A**) CEBwt, (**B**) CEBm3 and (**C**) CEBm7 in different buffers. Heating runs were performed between 10°C and 95°C; the temperature was increased by 0.2°C/min, and the absorbance was recorded at 260 and 295 nm. For each condition, the curve shown in solid (either corresponding to absorbance at 295 or 260 nm) was used to calculate the *T*_m_.

All UV-absorbance melting profiles at 295 nm are shown in Figure S3. The wild type CEB (CEBwt; *N* = 0) mainly formed a G4 structure in all buffers, and *T_m_* of G4s increased with potassium concentration (Figure S3a). A very similar behavior was displayed by CEBm1 (*N* = 1) (Figures 1A and S3d). Differently, the increased number of cytosines shifted the equilibrium towards the generation of hairpin structures in low potassium-contained buffers. CEBm3 (*N* = 1.67) predominately formed hairpins in low potassium concentration buffers (B1 and B3), while a G4 specific signature in the melting curves could be observed in high potassium concentration buffers (Figures 1B and S3f). For the sequences with a high number of cytosines (*N* = 4 for CEBm7) hairpins predominated in all buffers with very similar melting temperatures (Figures 1C and S3j). Since the melting profiles of CEBm2 in B3 (as well as CEBm3 in B4) were ambiguous, TDS were recorded to track the G4 formation of CEBm2 and m3 in these buffers. As shown in Figure S4a, the negative peak at 295 nm evidenced the formation of G4 structures (29); TDS of CEBwt in B3 and B4 were used as positive control for G4 folding (Figure S4b). The predominant conformation and *T_m_* are gathered in Table 2.

**Table 2.** *T*_m_ (°C) of CEB DNA strand series in different buffers^a^

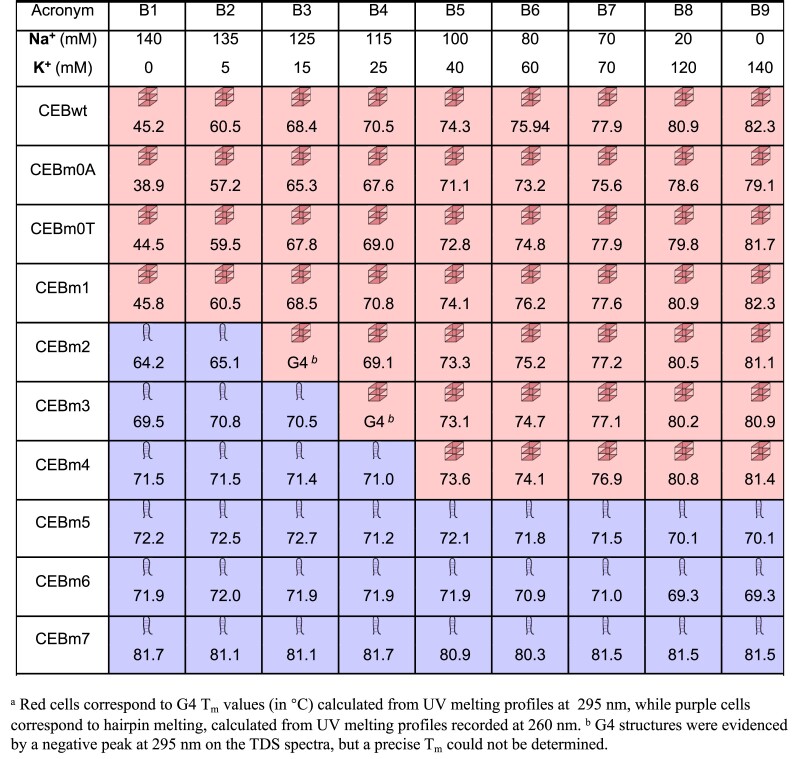

Under all buffer conditions (B1–B9), the predominant conformation adopted by CEBwt and CEBm1 was found to be a G4 structure. In contrast, the structures of the mutated sequences 2–4 depend on the specific buffer employed. In a low potassium concentration buffer (B1–B4, [K^+^] lower than 40 mM), CEBm1 (*N* = 1) predominantly adopts a G4 structure, even in the B1 buffer that does not contain potassium, but traces of a hairpin could be evidenced by the absorbance profile at 260 nm (Figure S3d). For the sequences with an increased number of cytosines, higher concentrations of potassium are required to observe G4 folding. CEBm4, characterized by *N* = 2, requires 40 mM potassium to fold into a G4 structure. Mutations 2–4 mainly fold into G4s at high potassium concentration (B4/B5 to B9, [K^+^] higher than 25 or 40 mM). Interestingly, under conditions where the quadruplex is the predominant fold (sequences CEBwt to CEBm4 in buffers B5–B9), its *T_m_* is nearly independent on the number of cytosines in the loops, and we found no significant difference in *T_m_* among the different mutated sequences. This observation illustrates that cytosines are not detrimental *per se* to G4 formation and that *T_m_* depends on buffer composition rather than on C content.

Loop composition is an element contributing to G4 folding. In a 1-nt propeller G4 loop, the substitution of a nucleotide with a single adenine reduces G4 melting by 6–8°C; differently, loops composed by cytosine, thymine, and guanine showed similar *T*_m_ (30,31). To confirm that central loop base substitutions did not strongly influence G4 intrinsic stability (besides altering G4-hairpin competition), especially in low potassium concentration conditions, we replaced some of the bases in the loops by adenines (CEBm0A) and thymines (CEBm0T). *T*_m_ of CEBm0T is very similar to the one of CEBwt, while CEBm0A shows a slightly lower thermal stability as compared to CEBwt and CEBm0T, in agreement with the literature (30,31). In the mutated C-rich sequences, we replaced one by one the bases present in the central loop by cytosines and observed that *T*_m_was not affected by loop base substitution. There is currently little information in the literature to explain how base composition in a long loop influences the stability of a G4. The small variation in G4 *T*_m_may be associated with the low fraction of adenines present in the central loop (2 adenines in the 9-nt loop (15). Our results suggested that cytosine substitutions in the central loop of CEBwt barely influence G4 thermal stability but increase the proportion of hairpins.

**Figure 2.**
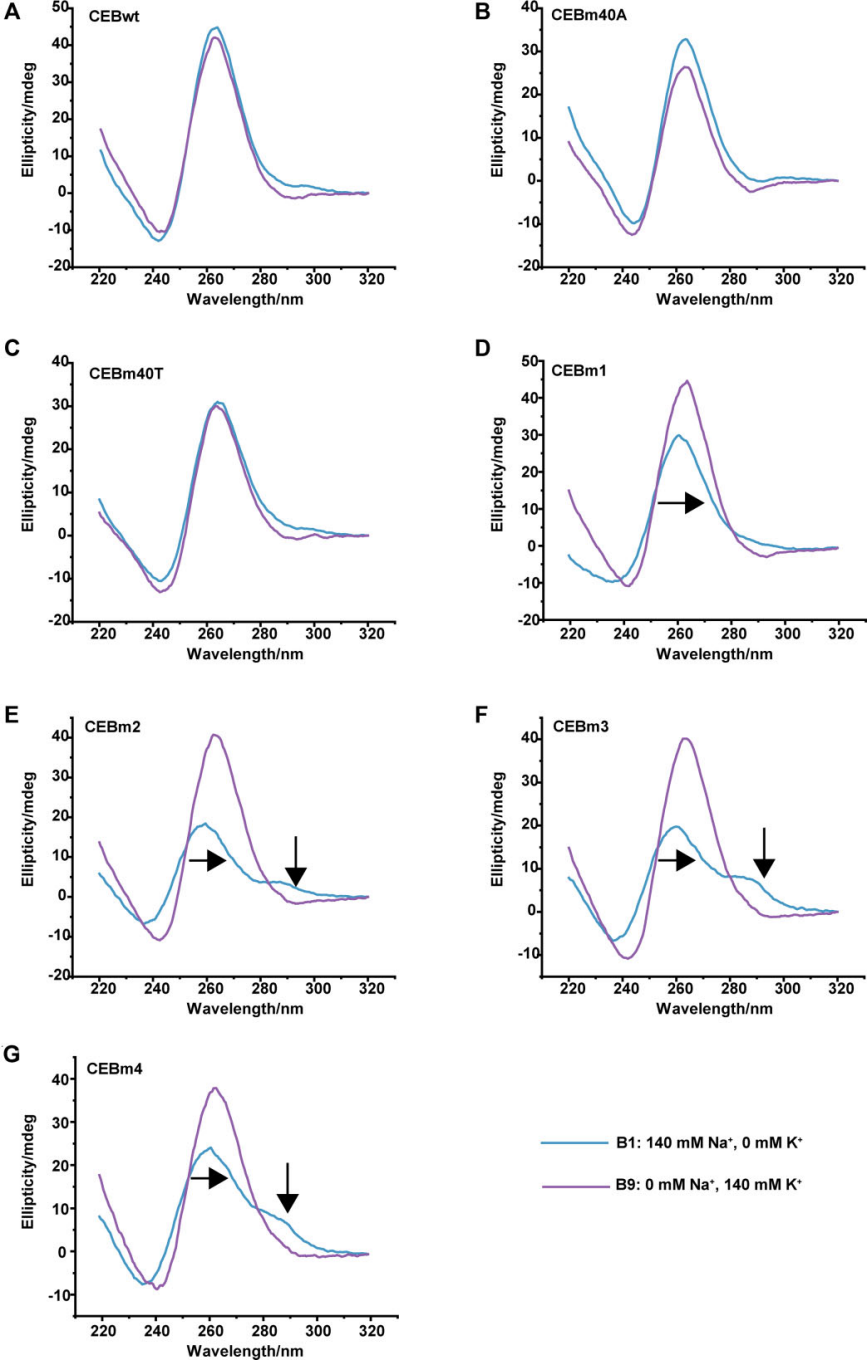
CD spectra of 5 μM CEBwt and its mutated versions in different buffers at 25°C. Arrows indicate peak shifts or peak intensity changes.

CD spectra were recorded to confirm topologies of formed G4 structures (Figures 2 and S5). CEBwt formed a parallel G4 in all buffers (Figure 2A), CEBm0A and CEBm0T also showed unambiguous G4 patterns in UV-melting experiments, and CD spectra evidenced that they form parallel G4 structures (Figure 2B, C). In contrast, CD spectra of mutated sequences CEBm1–CEBm4 exhibited specific peaks in low potassium concentration buffers (B1–B4), implying conformation conversion: CEBm1 in B1 showed a positive peak at 240 nm, which shifted to 242 nm in buffers containing higher [K^+^] (Figure 2D). CEBm2 and CEBm3 shifted the positive peak from 257 to 263 nm and 260 to 264 nm, respectively, at higher [K^+^] (Figure 2E, F). A 2 nm bathochromic shift was also observed for CEBm4 at higher [K^+^] buffer. Of note, CEBm3 and CEBm4 exhibited dichroic positive signals from 280 to 295 nm in low [K^+^] buffer; the intensity of the latter decreases gradually with increasing [K^+^] and could not be observed in high [K^+^] buffers (Figure 2F, G). These long wavelength maxima may be associated with the formation of G-C bp (32,33), confirming hairpin-G4 K^+^/Na^+^ dependent conversion.

Previous studies reported that metal ions may lead to G4 topology change. For example, pw17 d(G_3_TAG_3_CG_3_TTG_3_) folds into an antiparallel G4 in a buffer containing 5–70 mM Na^+^; however, the addition of <10 mM K^+^ buffer can switch the topology from antiparallel to parallel (34). The human telomeric G4 d[AGGG(TTAGGG)_3_] folds into an antiparallel G4 in 90 mM Na^+^ (PDB: 143D, (35) and into hybrid or parallel G4 in 50 mM K^+^ (PDB: 1KF1, (36). Loop composition may also lead to topology switching. L2T4 d(G_3_TG_3_T_4_G_3_TG_3_) forms an antiparallel G4 in 100 mM Na^+^, while L2A4 d(G_3_TG_3_A_4_G_3_TG_3_) folds into a parallel G4 in the same buffer (37). In contrast, in this work, CD analysis (Figure 2) demonstrates that whenever a G4 is formed by any of the CEBwt or mutant sequences, it remains parallel, independently of the potassium/sodium ratio. Therefore, the mutations of the loop sequence did not change G4 topology and minimally affect stability. The 1-nt loops impose a chain-reversal character for two of the three loops, severely restricting topological versatility.

When *N* increased to 3 or higher, CEBm5–CEBm7 predominately formed hairpins under all ionic conditions. In contrast to G4 structures and as expected for duplexes, their *T_m_* was not affected by changes in [K^+^]/[Na^+^] ratio but depended on the expected number of base pairs formed with each specific sequence (Figure S3h-j). Both CEBm5 and CEBm6 allow the formation of up to 9 G-C base pairs, yet with a different organization of loops and dangling-ends: CEBm5 is expected to adopt a hairpin structure with a relatively small central loop and a 6-mer dangling-end (Figure S6a). In contrast, CEBm6 was predicted to form a bilaterally symmetrical hairpin with a longer loop (Figure S6b). Previous study has shown that the presence of dangling ends minimally affect the hairpin stability (38), while loop size is an important element affecting hairpin stability. Generally speaking, a 4-nt loop is optimal for hairpin formation in 100 mM [Na^+^], and the stability of the hairpin decreases for longer loops, as larger loops are expected to have weaker intraloop hydrophobic interactions and stem-loop interactions (39–41). In our work, no significant difference in thermal stability was found between CEBm5 and CEBm6 (Figure S3h,i).

UV-melting results suggest that three ‘CCC’ motifs are enough to convert G4s to hairpins in all buffers, no matter their specific hairpin conformations. We then replaced the CEBm6 loop bases by cytosines, increasing *N* to 4. Unsurprisingly, CEBm7 prefers to form a hairpin (Figure S6c). As expected, compared to CEBm5 and CEBm6, CEBm7 has a *T_m_* 10°C higher, as G-C bp is more stable than A-T bp (Table 2).

### CEBm4 as a prototypical example of a Hairpin/G4 switch depending on buffer conditions.

UV-melting and CD spectra suggested that the structure of CEBm4 depends on the potassium/sodium ratio: CEBm4 mainly forms a hairpin in low [K^+^] conditions and a G4 structure in high [K^+^] buffers. To confirm this potassium/sodium-dependent structural transition, we performed a series of experiments to demonstrate that the structures of CEBm4 in B1 and B9 are completely different: CEBm4 adopting a hairpin in B1 and a G4 in B9.

*N*-Methyl mesoporphyrin IX (NMM) is a G4-specific fluorescence light-up probe (42,43) with a strong preference for parallel conformations (44). In the presence of CEBm4, the fluorescence of NMM does not change significantly in B1 as compared to the negative control (CEBm7), suggesting that CEBm4 is barely forming a G4 in these conditions. Conversely, the fluorescence signal raised in the B2 buffer, and this increase is even more pronounced in B3–B9 buffers containing higher [K^+^], implying that G4s exist under these conditions (Figure S7).

**Figure 3.**
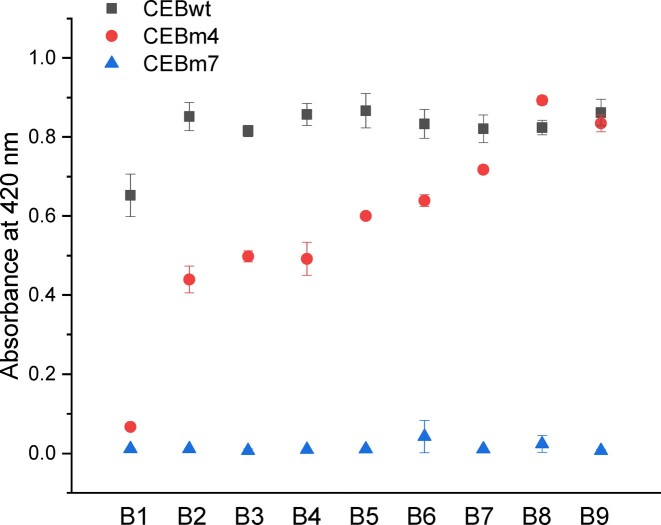
Absorbance at 420 nm resulting from H_2_O_2_-dependent oxidation of ABTS^2−^ to ABTS^•−^ by three different sequences (CEBwt, CEBm4 and CEBm7) in B1–B9 buffers.

We then used the peroxidase-like G4-DNAzyme activity assay to confirm the conformation transition of CEBm4. Although DNAzyme activity has rarely been used as a proof for G4 formation, it seems a relatively straightforward approach to test whether a sequence adopts a G4 fold or not, as most G4-based structures exhibit an enhanced catalytic ability. For example, Xiao *et al.* (45) followed G4 disruption produced by styrene oxide covalent binding thanks to the decrease in DNAzyme activity. Since the G4/hemin complex can catalyze the oxidation of ABTS^2-^(46) in the presence of H_2_O_2_, the formation of a G4 structure could be evidenced by the accumulation of the chromogenic oxidation product ABTS^•-^, which has specific absorbance properties. As shown in Figure 3, CEBwt display good catalytic activity under nearly all buffer conditions. In contrast, CEBm7 was completely unable to catalyze the oxidation reaction of ABTS^2-^even in B9 buffer, implying that CEBm7 was fully folded into a hairpin in all buffer conditions, in agreement with UV-melting results. CEBm4 is not able to catalyze the oxidation of ABTS^2-^in the absence of potassium (B1), since ABTS^•−^ absorbance at 420 nm is close to the one of CEBm7. Differently, the catalytic efficiency of the DNAzyme increased in B2-B4 buffers to reach levels comparable to CEBwt in B7-B9 buffers containing high [K^+^], implying that G4s may be partially or predominantly formed under these conditions. Hence, the DNAzyme activity confirms that the structure of CEBm4 can be converted from hairpin to G4 by changing the potassium/sodium ratio.

DNAzyme activity and NMM also suggest that G4s may be partially folded in low potassium concentration buffer (B2-B4), while G4 structure traces in these potassium-deficient environments could not be followed by UV-melting (Figure S3g). There are two possible reasons explaining these minor discrepancies: (i) UV absorbance at 295 nm may be too weak to be followed, as the preferred conformation of CEBm4 in potassium-deficient buffers is a hairpin, rather than a G4; (ii) Both hemin and NMM are G4 ligands and may therefore induce G4 structure formation and ‘rise up’ G4 population. In another word, G4 ligands may shift the hairpin/G4 equilibrium toward G4.

**Figure 4.**
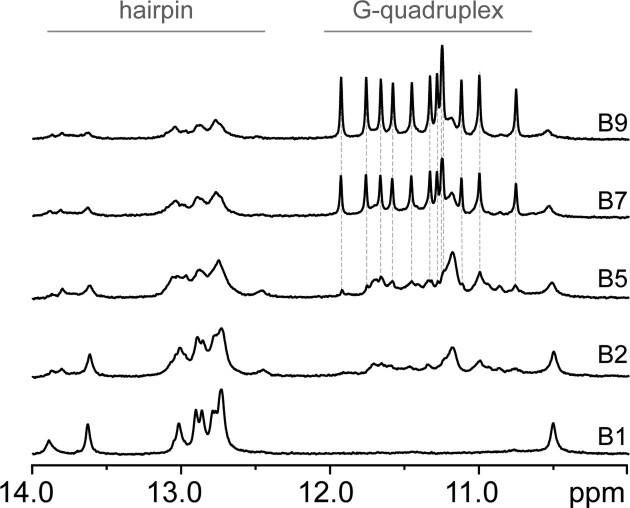
Imino regions of 1D ^1^H NMR spectra of CEBm4 under different ionic conditions (B1: 140 mM Na^+^/0 mM K^+^; B2: 135 mM Na^+^/5 mM K^+^, B5: 100 mM Na^+^/40 mM K^+^; B7: 70 mM Na^+^/70 mM K^+^; B9: 0 mM Na^+^/140 mM K^+^) in 10 mM lithium cacodylate (d7) buffer, pH 7.2 recorded at 25°C (the same set of spectra at 37°C are shown as Figure S9). B1 and B9 buffers are close to extra- and intra-cellular ionic conditions, respectively.

Finally, we performed 1D ^1^H NMR analysis of CEBm4 in five different buffers (Figure 4). In agreement with our expectations, imino region of 1D ^1^H NMR spectrum of CEBm4 in B1 shows signals between 12.0 and 14.0 ppm consistent with Watson-Crick base pair formation (47). Based on the sequence of CEBm4, we expected the formation of six Watson–Crick G-C base pairs in combination with additional A-T base pair(s). However, at 25°C we can observe signals between 12.6 and 13.1 ppm that belong to more than six G-C base pairs and suggest the presence of more than one hairpin structure in the solution (Figure 4). At 37°C, the number of imino signals of CEBm4 in B1 corresponds to six G-C base pairs (signals between 12.6 and 13.1 ppm) together with one A-T base pair (13.6 ppm) (Figure S8a). These Watson-Crick base pairs constitute the core of the hairpin. Additionally, we can observe two less intense signals at 13.9 and 10.5 ppm, respectively. The first signal at 13.9 ppm most probably belongs to A-T pair formed by A2 and T18, while the signal at 10.5 ppm might belong to A1-G19 base pair. The chemical shift of this predicted G-A base pair is in agreement with previously observed cases (48–50). These results indicate that the *hairpin A* is the predominant species of CEBm4 in B1, while the additional imino signals at 25°C most probably belong to minor population of *hairpin B* (or some similar structure based on G-C base pairs) concurrently present in the solution at lower temperature (Figure S8b).

Altogether, these signals confirm that CEBm4 exclusively forms a hairpin in B1, while in the buffers from B2 on we observe the formation of G4 in a [K^+^]-dependent manner (Figure 4). The imino signal pattern characteristic for parallel CEBwt G4 structure (grey dotted lines in Figure 4) was observed in buffers B5, B7, and B9, respectively. Concurrent observation of the proton resonances in the imino regions of 1D ^1^H NMR spectra of CEBm4 typical for hairpin and G4 structures, respectively, suggests the co-existence of both types of structures in these buffers, with G4 being predominant. A minor population of hairpin was observed even in B9 (estimated to be around 10%), however, the presence of sharp and nicely dispersed signals between 10.7 and 12.0 ppm, whose chemical shifts characteristic for G-quartets with Hoogsteen-type hydrogen-bonded guanines (51), support predominant G4 formation of CEBm4 in K^+^-based buffer.

### Hairpin/G4 molecular switching is slow

**Figure 5.**
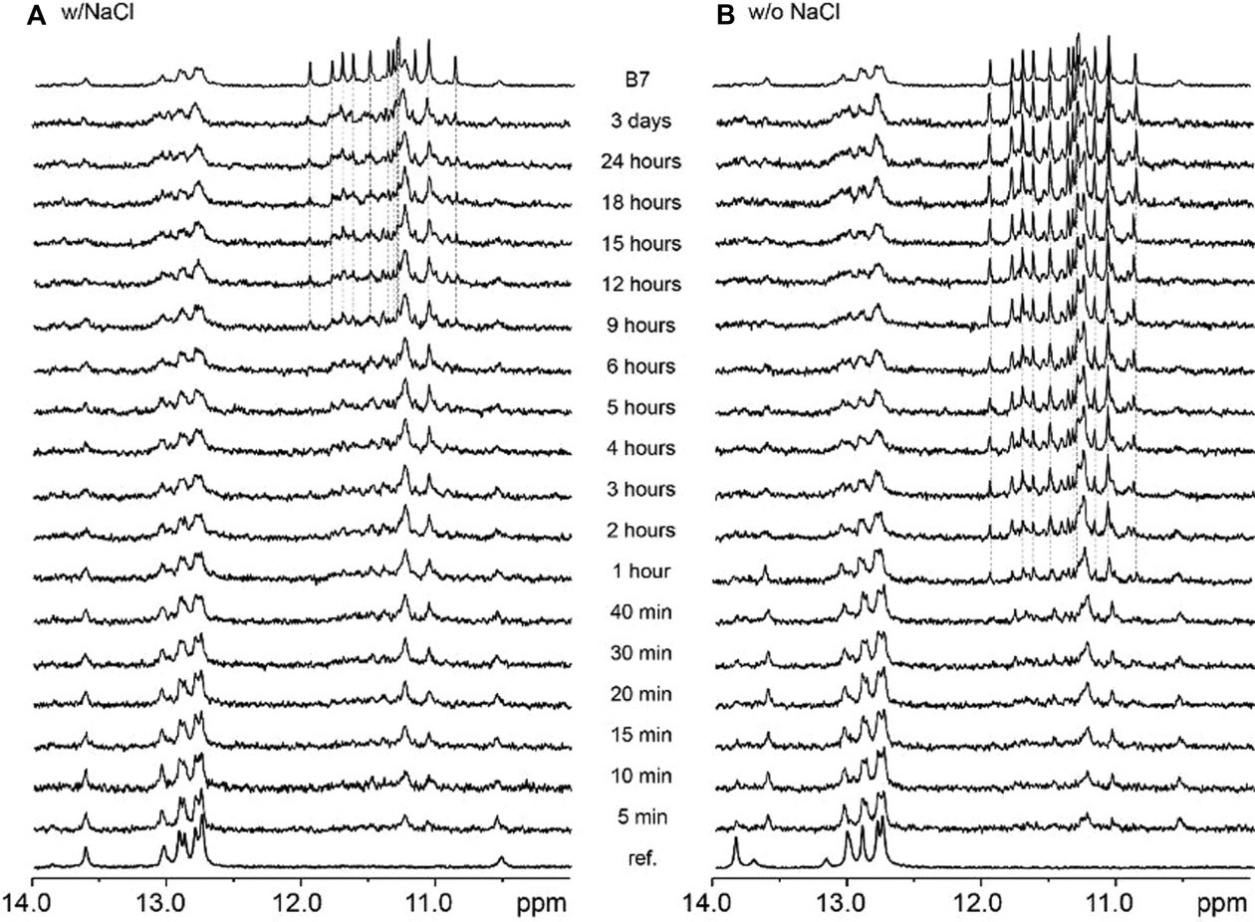
Imino regions of time-resolved 1D ^1^H NMR spectra of CEBm4 acquired at 37°C in lithium cacodylate buffer, pH 7.2, and (**A**) presence of 140 mM NaCl (w/NaCl) or (**B**) without salt (w/o NaCl), respectively. Both samples were diluted and KCl was added to final concentrations of 70 mM NaCl and 70 mM KCl (w/NaCl), or 70 mM potassium only (w/o NaCl), respectively. 1D ^1^H NMR spectra were recorded at different time points after KCl addition (from 5 min to 3 days). Time-dependent formation of G4 was monitored by the appearance of imino signals in the region between 10.5 and 12.0 ppm. CEBm4 annealed in B7 buffer (i.e. 10 mM lithium cacodylate (d7), pH 7.2 with concurrent presence of 70 mM NaCl and 70 mM KCl) is shown on top (‘B7’).

To determine how fast the CEBm4 would respond to changes in sodium/potassium balance, we performed time-resolved measurements using NMR (Figure 5) and CD spectroscopy (Figure S10). In both cases, we either started from the oligonucleotide prefolded in 10 mM lithium cacodylate buffer with 140 mM NaCl (w/NaCl), or without NaCl (w/o NaCl), respectively. The oligonucleotide was then diluted, and KCl was added to final concentrations of 70 mM NaCl and 70 mM KCl (w/NaCl) or 70 mM potassium only (w/o NaCl), respectively. Time-resolved NMR and CD experiments indicate (Figures 5 and S10) that the addition of K^+^ eventually leads to G4 formation, as shown by the appearance of imino peaks in the region between 10.5 to 12.0 ppm, or an increase in ellipticity at 260 nm, respectively.

However, as shown by ^1^H NMR experiments the kinetics of the interconversion between the two forms (hairpin and G4) appears to be rather slow (Figure 5). While in w/o NaCl sample signals, typical for G4 were observed approximately after one hour of KCl addition, the conversion in w/NaCl was far from complete even after 3 days (Figure 5A). The w/NaCl sample remained highly polymorphic as indicated by several overlapping signals in the region between 10.5 and 12.0 ppm. Although in the presence of the same ratio of sodium/potassium ions, w/NaCl, even after 3 days after KCl addition, did not display the same imino region in comparison to the one obtained after proper annealing in the B7 buffer and population of CEBm4 G4 remained low the entire period of the experiment. This illustrates that pre-folding of the hairpin in NaCl acts as a kinetic trap at 37°C, impeding the conversion to thermodynamically favored species, CEBm4 G4.

On the other hand, hairpin to G4 conversion was faster when no NaCl was present in the solution (w/o NaCl) (Figure 5B). Although annealed in low salt conditions, (no counter ions besides Li^+^ from lithium cacodylate buffer present), w/o NaCl formed a hairpin, which was not identical to the one in the presence of NaCl. Upon the addition of KCl, the conformation of the hairpin formed in solution without NaCl switched to the same hairpin as formed in the presence of NaCl. From here on the imino regions of both samples looked comparable up to 40 min after KCl addition. After that, we started to observe signals typical for forming parallel CEBm4 G4 in w/o NaCl. The population of CEBm4 G4 in solution without NaCl rises with time and becomes predominant, while other G4 folds represent only negligible populations. The kinetics of G4 formation of w/o NaCl is faster than w/NaCl: this is most likely because the starting hairpin is more stable in the presence of salt, and that it needs to be unfolded, at least partially, to allow G4 formation. The energetic barrier for this opening becomes a rate-determining step for G4 formation, explaining why we could not observe full conversion to G4 in our time-frame (3 days for *in vitro* NMR). Note, however, that G4 formation of a G4 with a long loop is not very fast *per se*, even in the absence of any competing structure, as shown by A.T. Phan and colleagues (52).

Both experiments illustrate that the CEBm4 system can respond to changes in sodium/potassium balance. Still, with strong kinetic inertia: while this probe may be used to measure equilibrium concentrations in these cations, it cannot monitor rapid changes in their balance. This experiment also illustrates that forming a competing duplex hairpin structure can act as a kinetic trap and further delay G4 formation.

### Intracellular Na^+^/K^+^ are decisive factors for modulating hairpin/G4 equilibrium in the intracellular environment.

**Figure 6.**
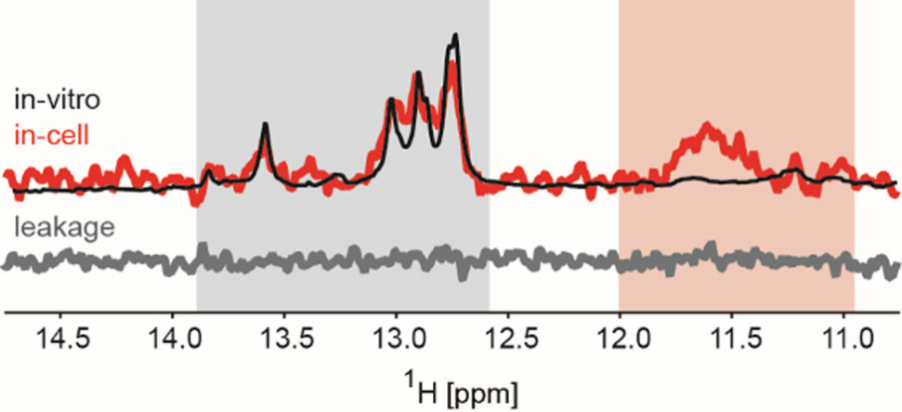
The overlay of imino regions of 1D ^1^H NMR spectrum of CEBm4 acquired in the electroporation buffer (140 mM NaCl, 5 mM KCl, 5 mM MgCl_2_, NaPOi, pH 7.2) [in black] and in-cell 1D ^1^H NMR spectrum acquired within a time window of ∼30 min on the suspension of the HeLa cells transfected with CEBm4 [in red] at 37°C. The corresponding region of the 1D ^1^H NMR spectrum of the extracellular fluid taken from the sample after the in-cell NMR spectrum acquisition recorded at 37°C is shown in gray (leakage control). The gray and red box highlights the spectral region specific for hairpin and G4 imino signals, respectively.

The CEBm4 data showed that hairpin to G4 equilibrium is sensitively modulated by sodium/potassium balance under simplistic buffer conditions. To assess whether and how this transition is modulated in the complex intracellular environment, which next to K^+^/Na^+^ ions include other factors known to modulate structural equilibria involving G4/duplex (or hairpin) DNA (*e.g*.molecular crowding), we attempted to assess the behavior of CEBm4 in the intracellular space of living human HeLa cells using in-cell NMR spectroscopy, as recently done for i-DNA (53). CEBm4 was pre-folded in a buffer resembling the composition of the extracellular space (140 mM NaCl, 5 mM KCl, 5 mM MgCl_2_, NaPOi, pH 7.2). As demonstrated by the corresponding NMR spectrum displaying imino signals in a region characteristic for forming Watson-Crick base-pairs (12–14 ppm) (Figure 6, black line), CEBm4 folds into hairpin under these conditions. The CEBm4 hairpin was subsequently electroporated into the intracellular space of living HeLa cells. This process did not compromise the cells’ viability/membrane integrity (Figure S11a). The confocal image inspection of the transfected cells corroborated nuclear oligonucleotide localization (Figure S11b). 1D ^1^H in-cell NMR spectrum was acquired within a time window of ∼30 min on the suspension of the electroporated cells at physiological temperature (37°C). The resulting in-cell NMR spectrum displayed characteristic imino signals from hairpin (12–14 ppm) as well as several signals in a region typical for imino protons involved in Hoogsteen base pairs, including a broad unresolved signal consistent with the formation of a G4 polymorph (10–12 ppm) (Figure 6, red line). Notably, the ratio of the hairpin to G4 signals in the in-cell NMR spectrum of CEBm4 was similar to that in the corresponding *in vitro* NMR spectrum acquired in the presence of NaCl ∼20–30 min post addition of KCl (Figure 5A). The in-cell NMR data thus indicate that refolding of CEBm4 hairpin into a G4 in the intracellular space proceeds with comparable kinetics as those observed *in vitro*, suggesting that sodium/potassium balance in the intracellular space is the primary driving factor modulating the hairpin/G4 equilibrium in the intracellular space.

## Discussion and conclusion

**Figure 7.**
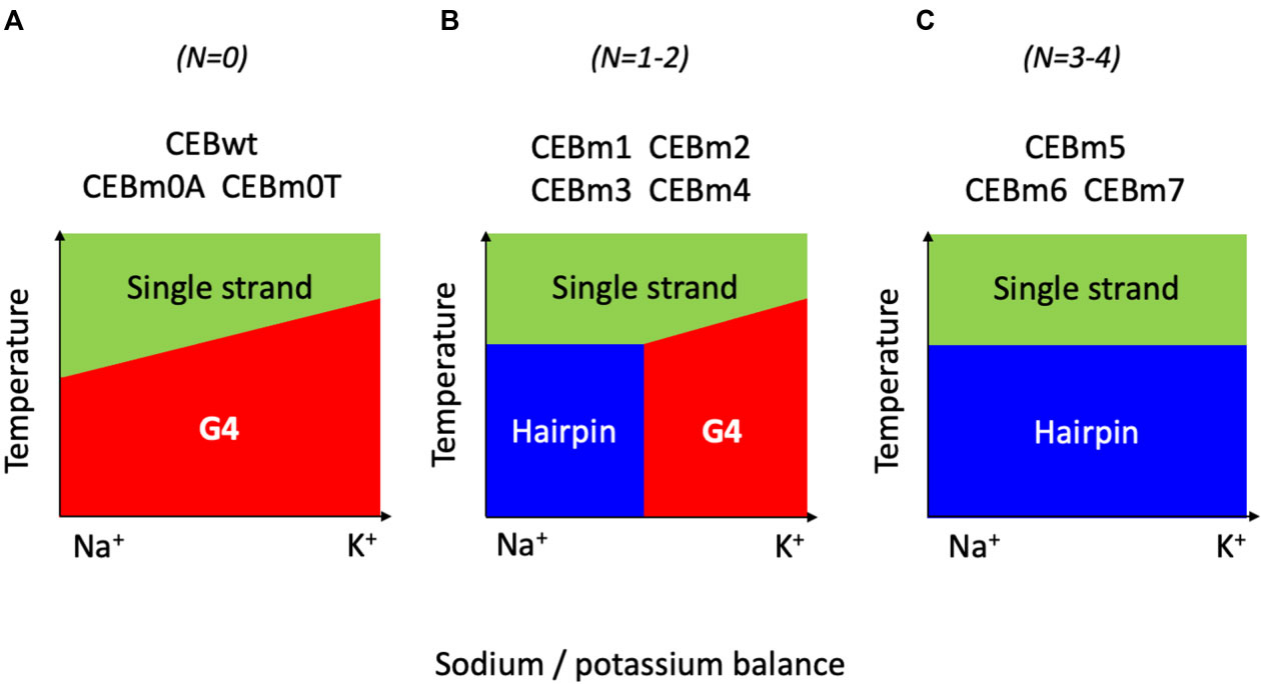
Schematic Phase diagrams showing the predominant species (single-strand, Hairpin or G4) as a function of temperature and sodium/potassium ratio. Three situations were encountered: (**A**) very few cytosines are present in the primary sequence, and the G4 always predominates at lower temperatures; *T*_m_ depends on K^+^ concentration. (**B**) Intermediate case, in which a limited number of CCC runs allows the Hairpin to predominate at low temperature and low potassium concentration, while a G4 is formed once a certain K^+^ concentration threshold is reached (the exact threshold being sequence-dependent). (**C**) C-rich sequences do not adopt a G4 fold, no matter the potassium concentration: the Hairpin predominates at low temperature; its *T*_m_ depends on the exact sequence but is independent on Na^+^/K^+^ ratio.

The *T*_m_ of the CEB sequences measured in different buffers revealed that the presence of cytosines in G4 loops does not decrease G4 stability but rather increases the probability of forming a competing structure, such as an intramolecular hairpin. In detail, three continuous cytosines in the central loop barely influence G4 formation under all potassium/sodium conditions tested. With an increasing number of cytosines, the hairpin starts to be the predominant structure under low potassium concentrations (0–40 mM), while G4 folding still occurs in the presence of sufficient potassium ion concentration (40–140 mM). The *T*_m_ values of these G4 structures only depend on the specific potassium/sodium ratio, rather than on the specific cytosine-content of the mutated sequences. When the number of ‘CCC’ runs further increases to three, the CEB mutated sequence predominately folds into a hairpin under all ionic conditions. Differently, *T*_m_ values of duplexes are barely affected by changes in [K^+^]/[Na^+^] ratios and only depend on the primary sequence (GC content). These properties are summarized in the three schematic phase diagrams shown in Figure 7.

Overall, these results illustrate that hairpin-quadruplex competition is a relevant phenomenon for the folding of G-rich sequences also containing runs of cytosines. Hairpin formation can act as a competing structure, especially in sodium-rich conditions. This is especially relevant when considering aptamer sequences to be used in the extracellular environment, where sodium predominates. Furthermore, even if the hairpin is not the thermodynamically favored species, its relatively fast formation may further delay the formation of a G4. Interestingly, the in-cell NMR data confirm the relevancy of *in vitro* measurements and derived predictions related to G4 formation in cells.

Possible hairpin formation was already taken into account by G4Hunter when predicting G4 propensity: runs of consecutive cytosines impose a penalty on the score, as shown in Table 1. This penalty does not mean that cytosines are detrimental *per se* to G4 formation, as confirmed in Table 2 by the nearly sequence-independent *T_m_* values of CEBwt to CEBm4 in the B5-B9 buffer. It rather reflects the possibility of adopting other folds, such as hairpins. G4Hunter scores vary between 1.5 for the parent wild-type sequence and 0 for CEBm7. Table 2 illustrates that while CEBm4 can form a G4 under intracellular conditions (high K^+^), CEBm5 cannot. With respective G4Hunter scores of +0.69 and +0.32, this would indicate that, with the current parameters, the threshold value for stable G4 formation in K^+^ should be between these two numbers. However, most sequences with scores in that range do not form G4 (*unpublished results* and (54)). This observation tends to indicate that the penalty we impose due to the presence of cytosine runs may be slightly too severe: by choosing a ‘–2’ or ‘–1.5’ penalty for each C in a CCC run (instead of –3, as currently calculated (23), the G4Hunter score of CEBm4 would be increased to 0.92 or 1.04, *i.e*. values that would be more in line with possible G4 formation. In any case, we expect that data collection on ‘borderline’ cases should help refine predictions and better understand G4 formation rules.

## Supplementary Material

gkad1073_Supplemental_File

## Data Availability

All data are available in the manuscript and supplementary files.
