# Supplementary material for: A sodium/potassium switch for G4-prone G/C-rich sequences
Source: Nucleic Acids Res. 2023 Nov 20;52(1):448–61. doi: 10.1093/nar/gkad1073 (PMC10783510; doi:10.1093/nar/gkad1073)
Supplement: gkad1073_Supplemental_File [file gkad1073_supplemental_file.pdf]

A sodium / potassium switch for G4-prone G/C-rich sequences

Supplementary Information

Yu Luo[1,4], Martina Lenarčič Živković[2,3], Jiawei Wang[1]
Jan Ryneš[2], Silvie Foldynová-Trantírková[2], Lukáš Trantírek[2],
Daniela Verga[4,5*], & Jean-Louis Mergny[1*]

1. Laboratoire d'Optique et Biosciences, Ecole Polytechnique, CNRS, Inserm, Institut Polytechnique de Paris, 91128 Palaiseau, France;
2. Central European Institute of Technology, Masaryk University, 625 00 Brno, Czech Republic;
3. Slovenian NMR Centre, National Institute of Chemistry, SI-1000 Ljubljana, Slovenia;
4. CNRS UMR9187, INSERM U1196, Université Paris-Saclay, F-91405 Orsay, France;
5. CNRS UMR9187, INSERM U1196, Institut Curie, PSL Research University, F-91405 Orsay, France.

Contents :

- *Table S1*
- *Figures S1-S11*
- *Additional references*

Table S1. Numbers of PQS found in human genome (hg19) (Database: GSE133379 [1])

Chromosome	Nos of PQS	PQS per kbp	Nos of 'CCC' contained PQS	Frequency of 'CCC' in PQS	Nos of two 'CCC's contained PQS
Chr 1	134804	0.54	13779	0.10	1068
Chr 2	110685	0.46	10124	0.09	671
Chr 3	79202	0.40	6412	0.08	424
Chr 4	60491	0.32	5138	0.08	357
Chr 5	70948	0.39	5924	0.08	391
Chr 6	68177	0.40	5358	0.08	369
Chr 7	78345	0.49	7823	0.10	597
Chr 8	64136	0.44	5977	0.09	447
Chr 9	69477	0.49	7659	0.11	627
Chr 10	71686	0.53	7336	0.10	561
Chr 11	81053	0.60	8566	0.11	675
Chr 12	64896	0.48	6131	0.09	565
Chr 13	32452	0.28	2737	0.08	244
Chr 14	46353	0.43	4687	0.10	346
Chr 15	49090	0.48	4953	0.10	309
Chr 16	65359	0.72	7996	0.12	708
Chr 17	74609	0.92	8919	0.12	682
Chr 18	30684	0.39	2669	0.09	199
Chr 19	75540	1.28	10294	0.14	902
Chr 20	46930	0.74	5162	0.11	440
Chr 21	19109	0.40	2381	0.12	226
Chr 22	43195	0.84	5930	0.14	461
Chr X	59843	0.39	4384	0.07	289
Chr Y	9289	0.16	781	0.08	48
Sum	1506353	-	151120	-	11606

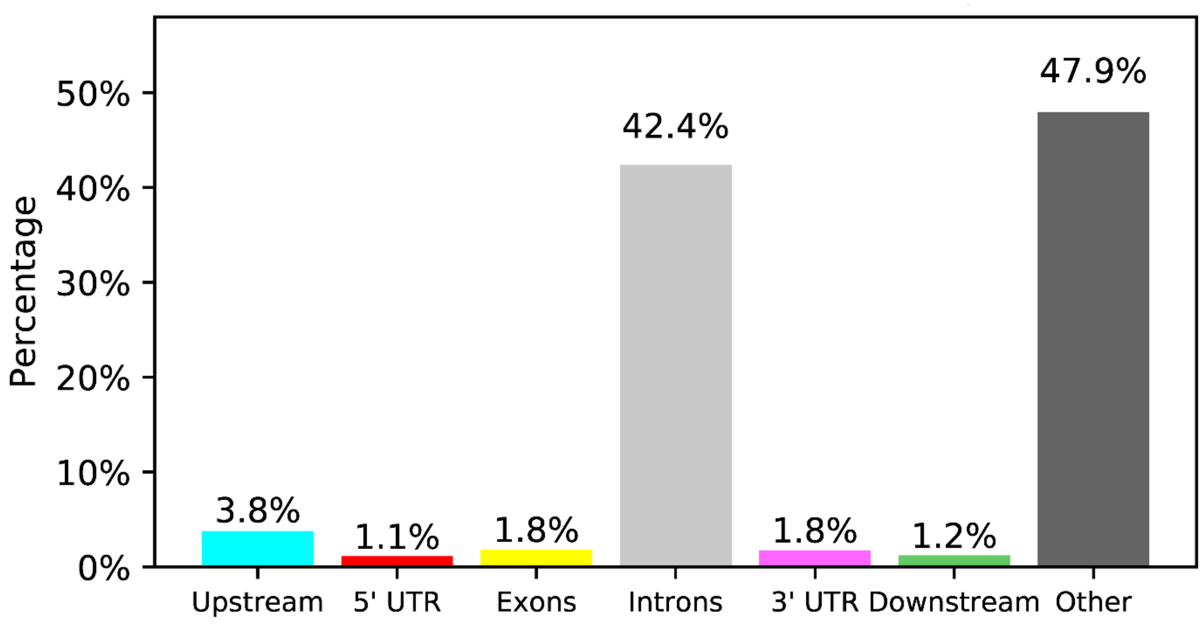

Figure S1. Genomic location and distribution of PQS containing a single 'CCC'-motif.

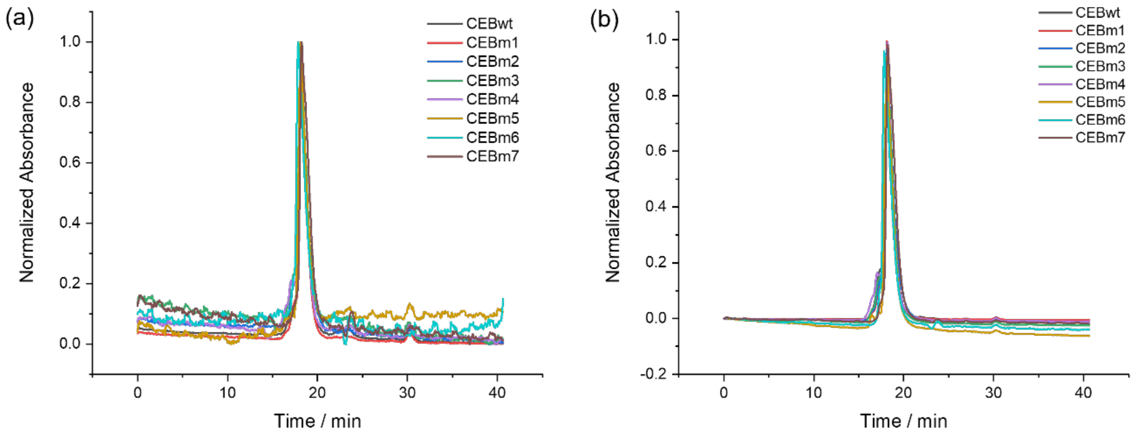

Figure S2. Analysis of the molecularity of folded species according to SE-HPLC. Normalized absorbance of mutations at (a) 10 mM LiCaco (pH = 7.2) buffer with 140 mM NaCl and (b) 10 mM LiCaco (pH = 7.2) buffer with 140 mM KCl. A single predominant species is found for all sequences, matching the peak obtained for CEBwt, previously determined to be a monomolecular species [2].

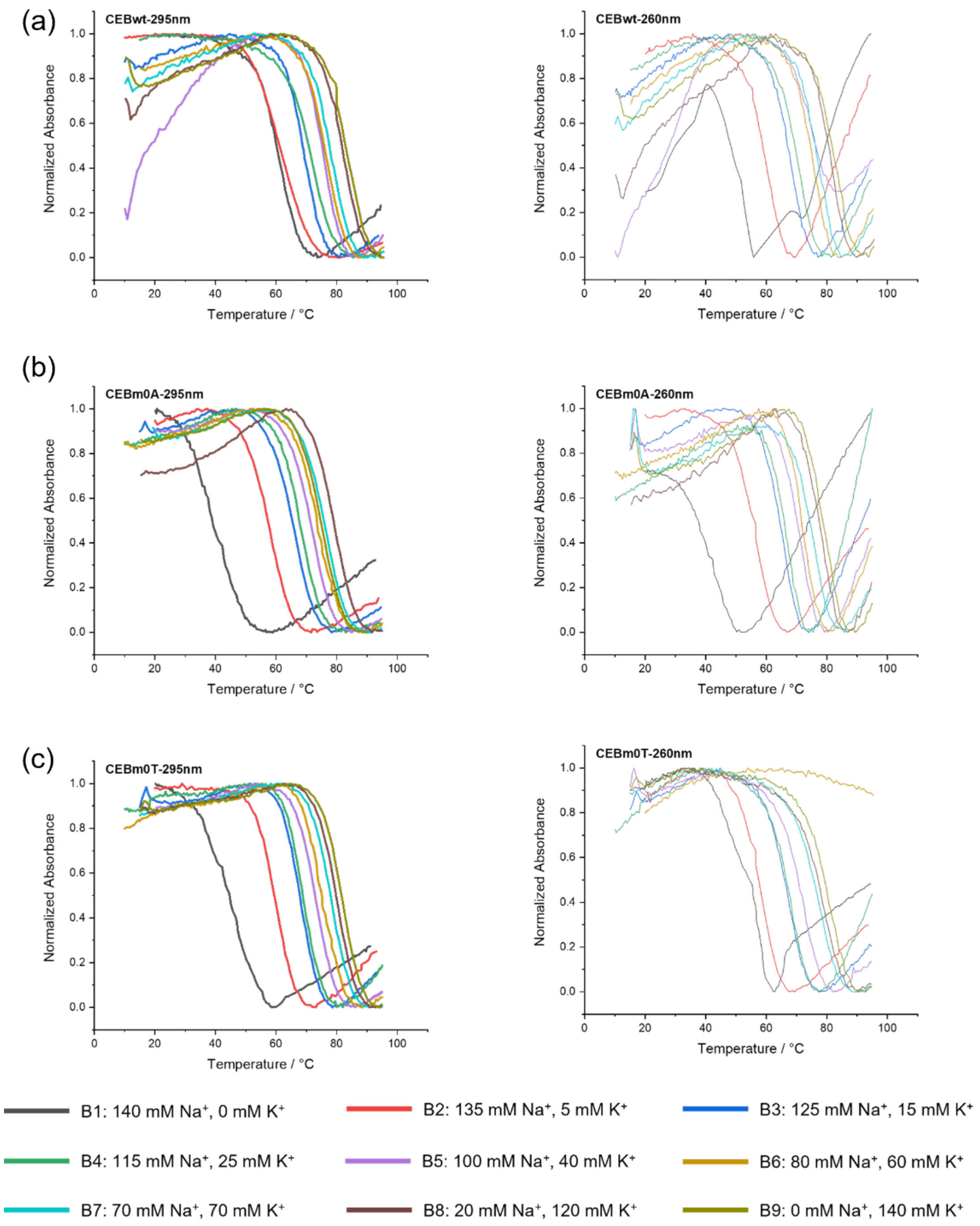

Figure S3. *To be continued to the next page.*

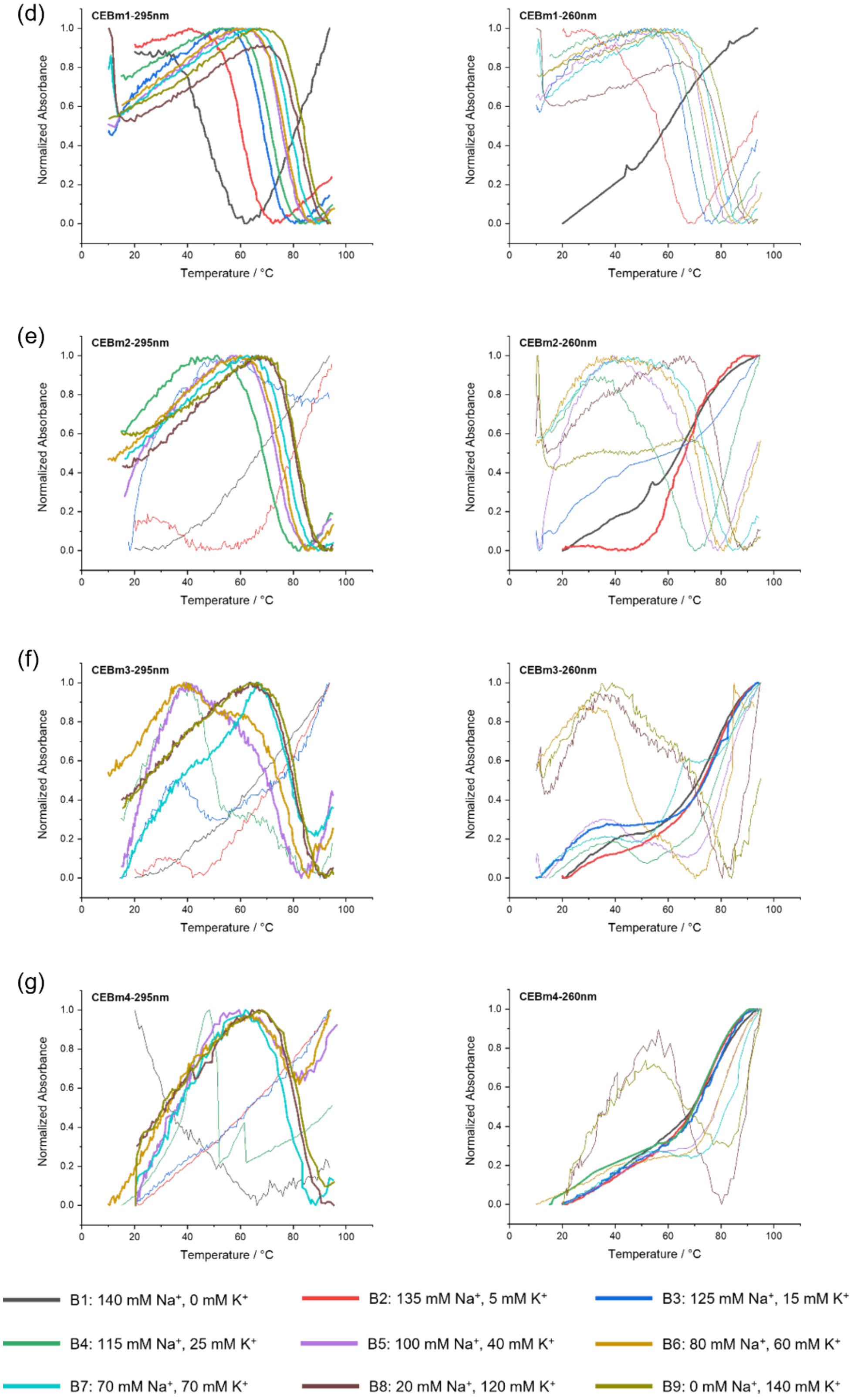

Figure S3. *To be continued to the next page.*

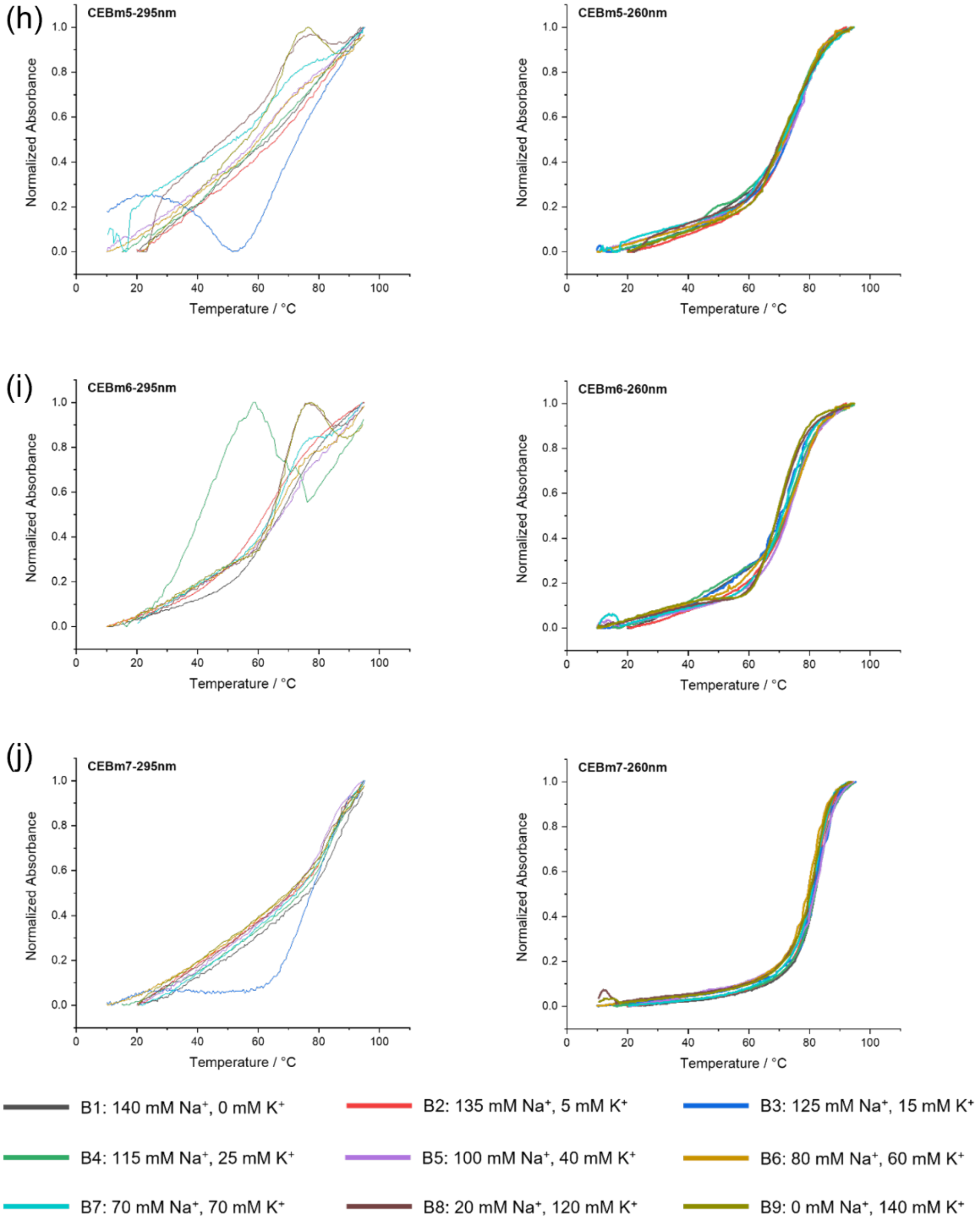

Figure S3. Normalized UV-melting curves of 5 µM CEB mutations in different buffers. Heating runs were performed between 10 °C and 95 °C, and the temperature was increased by 0.2 °C per minute, and the absorbance was recorded at 260 and 295 nm. Curves shown in bold were used to calculate the T_m.

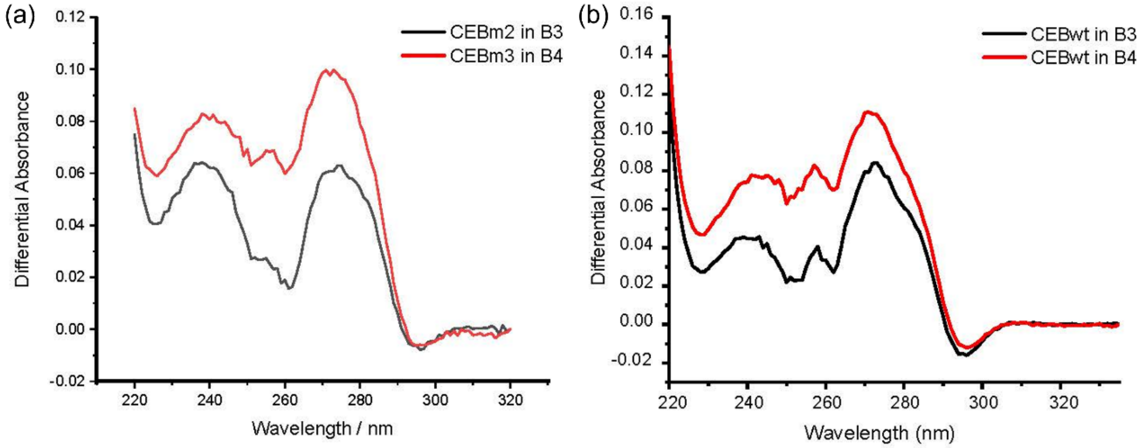

Figure S4. Thermal differential spectra (TDS) of 5 µM (a) CEBm2 in B3 buffer and CEBm3 in B4 buffer; (b) CEBwt in B3 and B4 buffers. TDS corresponds to the arithmetic difference between the initial (25 °C) and second (95 °C) spectra. B3 buffer (125 mM NaCl, 15 mM KCl in 10 mM LiCaco, pH = 7.2); B4 buffer (115 mM NaCl, 25 mM KCl in 10 mM LiCaco, pH = 7.2).

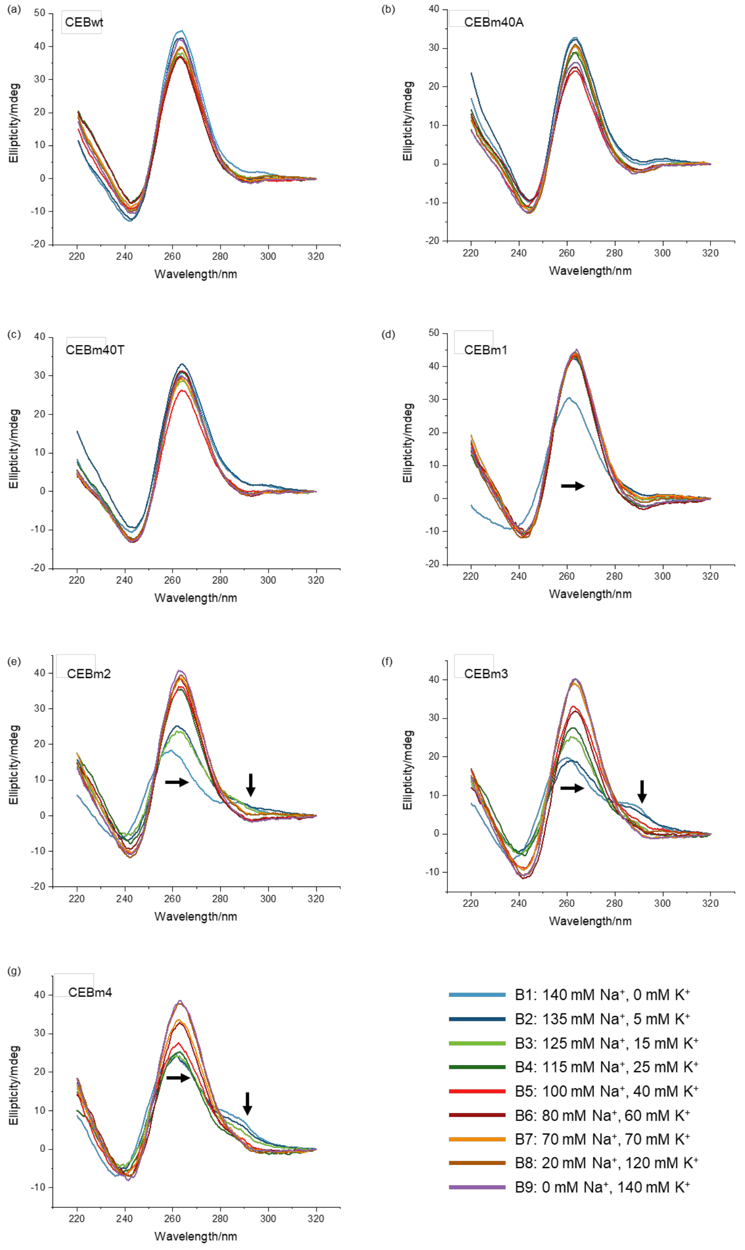

Figure S5. CD spectra of 5 μM CEBwt and its mutated versions in different buffers at 25 °C. Arrows indicate peak shifts or peak intensity changes.

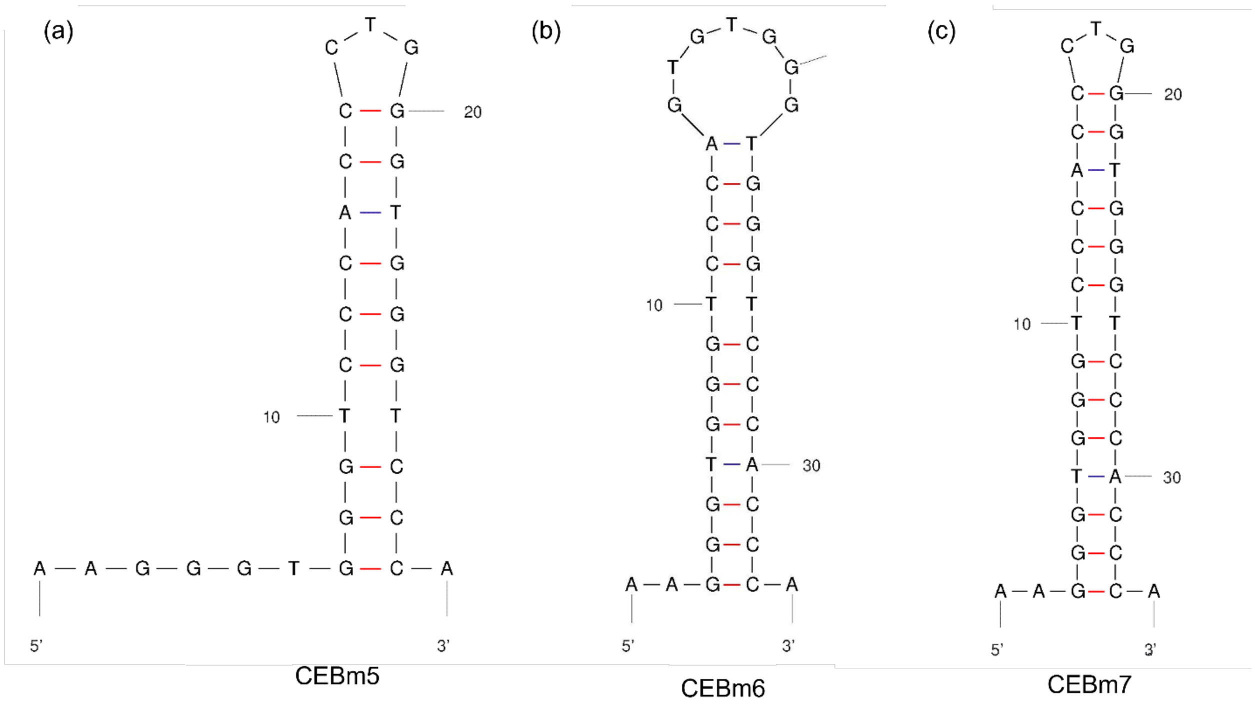

Figure S6. The hairpin models of CEBm5, CEBm6 and CEBm7 according to UNAFold [3].

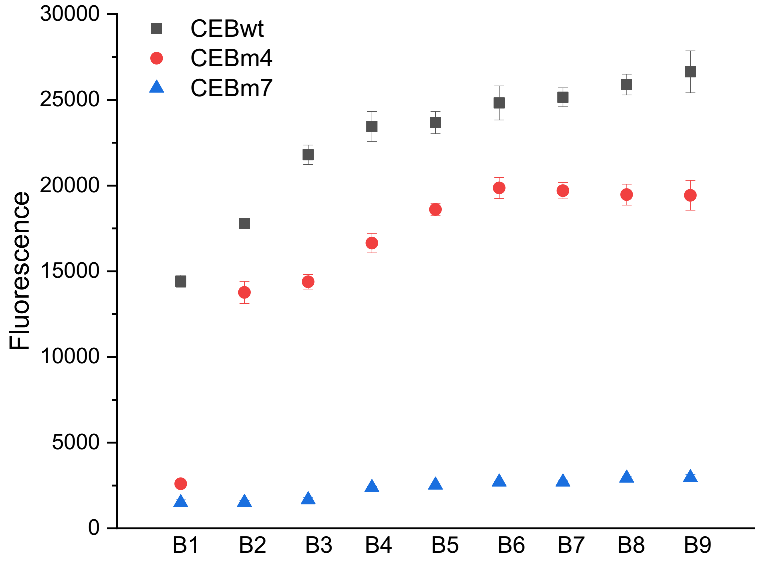

Figure S7. 2 µM NMM staining of structures generated by 3 µM of CEBwt, CEBm4, and CEBm7 in different buffers. Fluorescence was immedately measured after NMM addition.

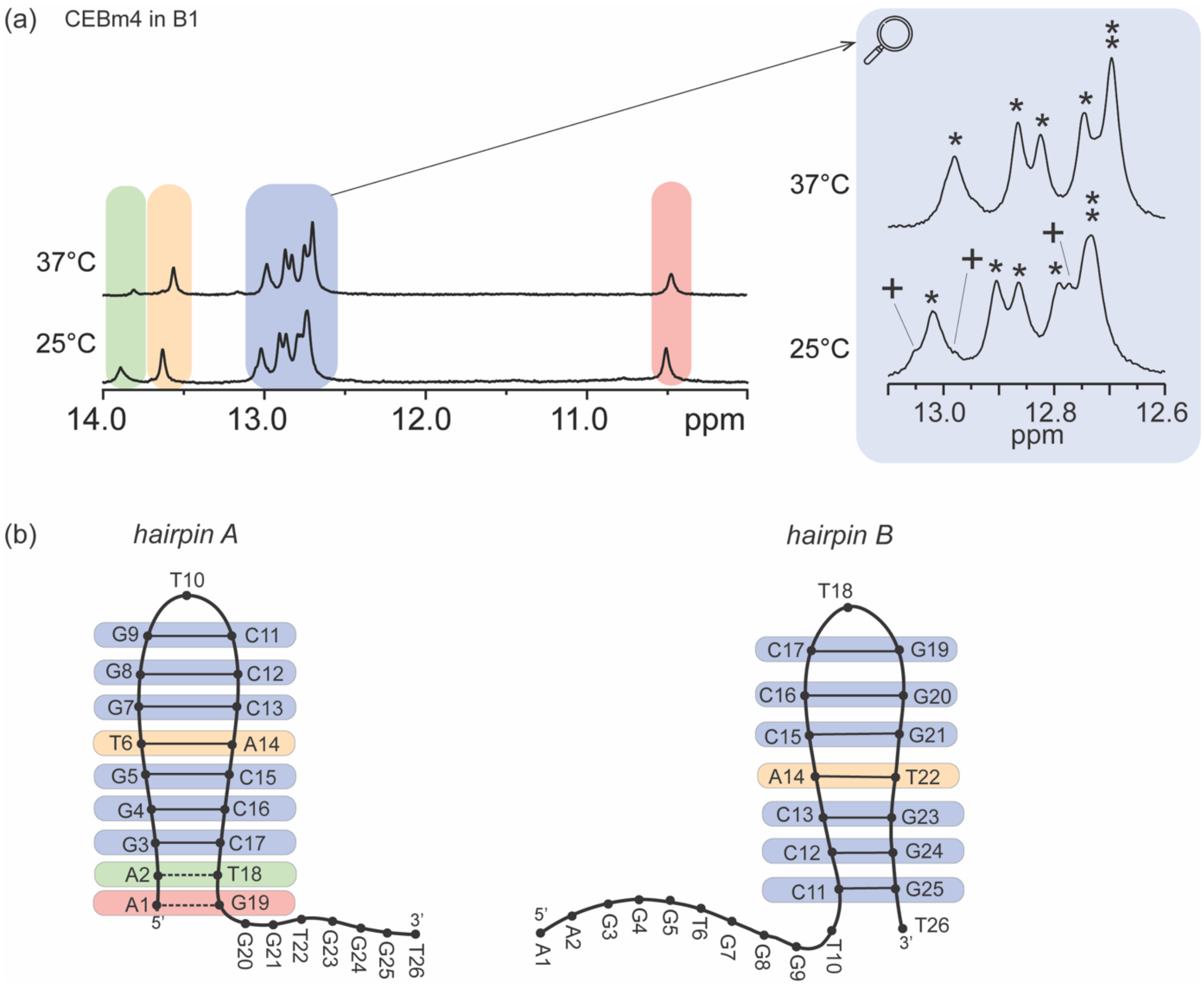

Figure S8. (a) Imino regions of 1D ^{1}H NMR spectra of CEBm4 recorded at 25 °C and 37 °C, respectively. (b) Proposed hairpin conformations of CEBm4. The overall spectra' appearance is consistent with the hairpin A model, identified as the major species at both temperatures. Note: At 25 °C, in the region between 12.6 to 13.1 ppm, aside from the six expected signals from G-C base pairs (*), there were additional signals (+), signifying the coexistence of a major conformation with minor species at this temperature (blue inset in (a)).

.

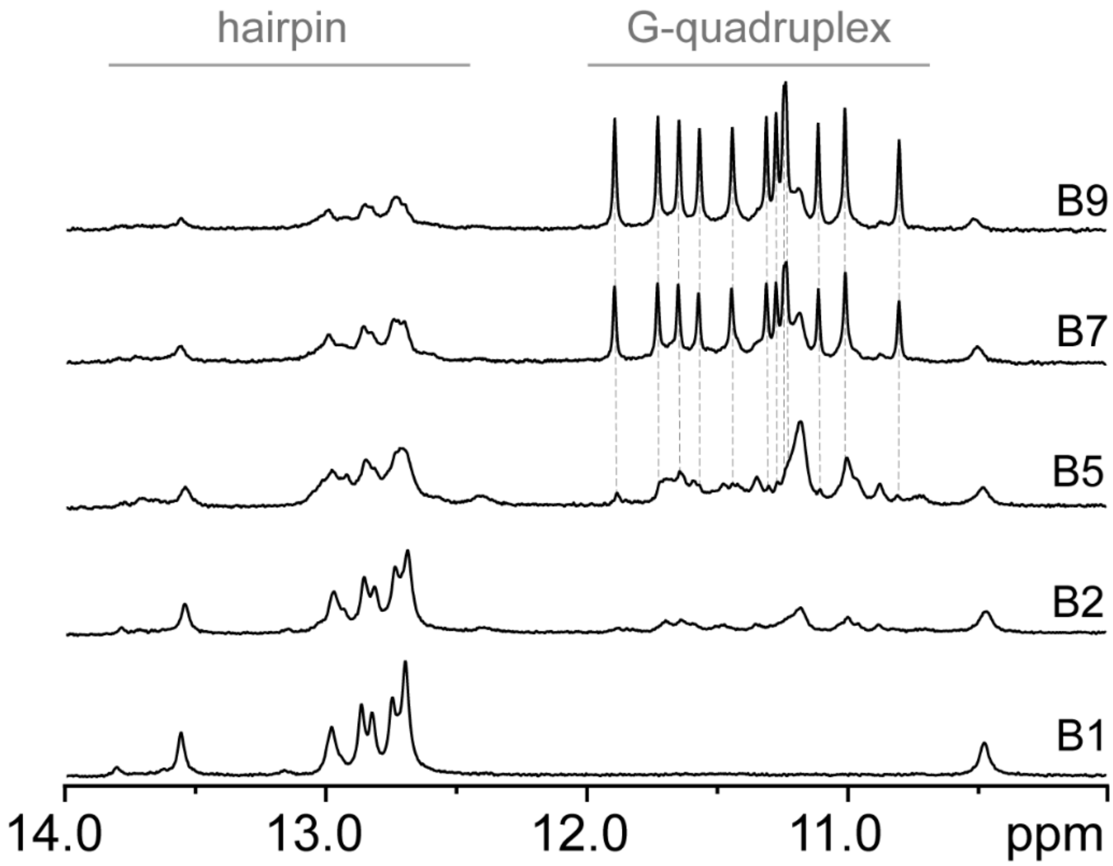

Figure S9. Imino regions of 1D ^{1}H NMR spectra of CEBm4 under different ionic conditions (B1: 140 mM Na^+ / 0 mM K^+; B2: 135 mM Na^+ / 5 mM K^+, B5: 100 mM Na^+ / 40 mM K^+;B7: 70 mM Na^+ / 70 mM K^+; B9: 0 mM Na^+ / 140 mM K^+) at 37 °C (the same set of spectra at 25 °C are shown as Figure 4). B1 and B9 buffers are close to extra- and intra-cellular ionic conditions, respectively.

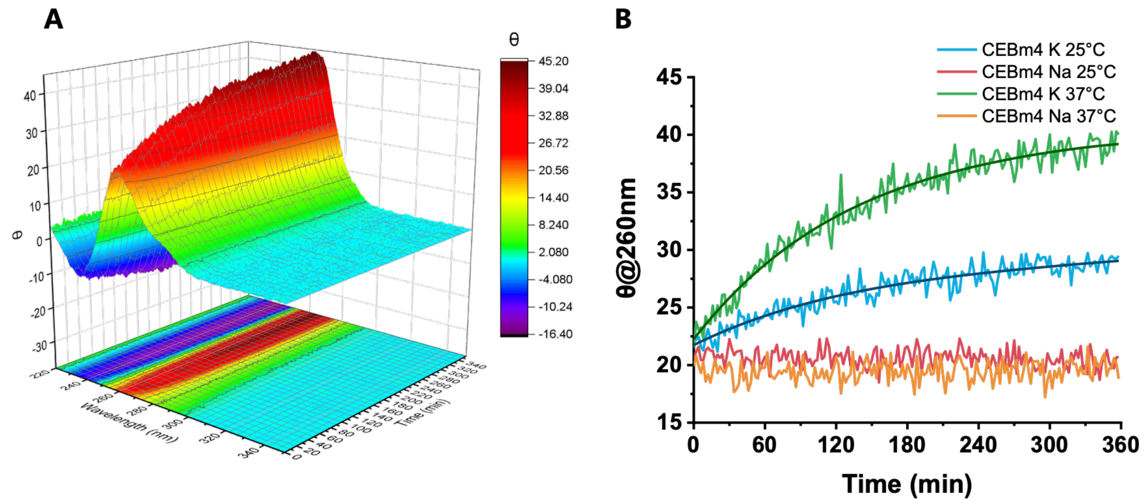

Figure S10. (A) Time dependent CD spectra recorded upon dilution in K^+ buffer. Starting solution: 70 µM CEBm4 in 140 mM Na^+ at 37 °C. After diluting the solution to a final concentration of 5 µM CEBm4 in 10 mM Na^+ + 130 mM K^+, a CD spectrum is recorded every 2 minutes over a period of 6 hours. (B) Ellipticity at 260 nm as a function of time upon dilution in K^+ or Na^+ buffer at 37 °C and 25 °C. The half conversion times of CEBm4 in K^+ buffer at 37 and 25 °C are 102 and 350 minutes, respectively.

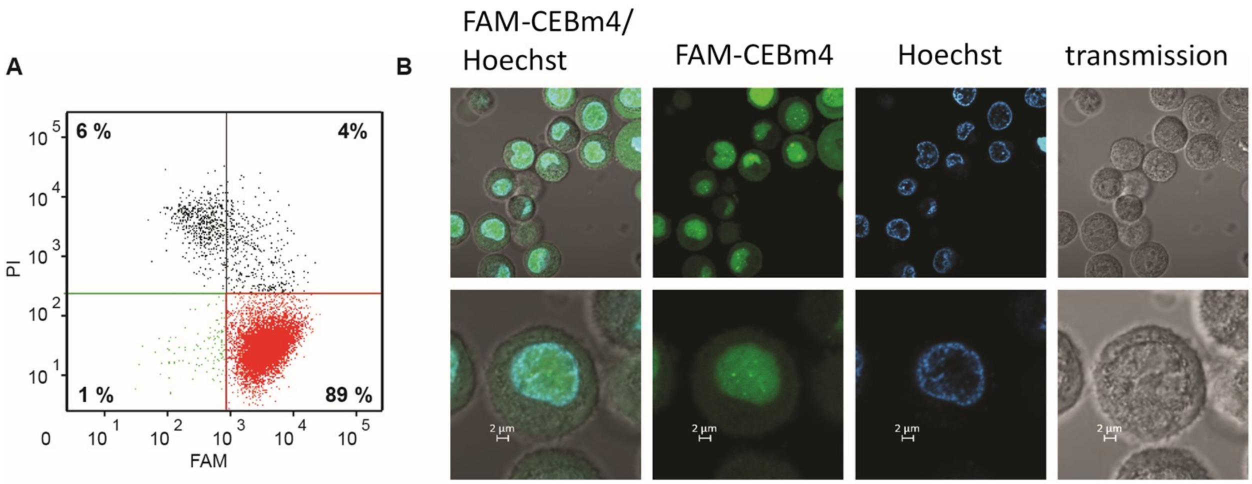

Figure S11. (A) Double-staining (DNA-PI/DNA-FAM) FCM analysis of HeLa cells transfected with (FAM-)CEBm4. The FCM plot indicates the percentages of viable nontransfected cells, viable CEBm4 DNA-containing cells, dead/compromised nontransfected cells, and dead/compromised cells transfected with CEBm4 in the bottom-left, bottom-right, top-left, and top-right quadrants, respectively. (B) Confocal microscopy images of HeLa cells transfected with (FAM-)CEBm4 and stained with Hoechst 33342. Green and blue colors indicate the localization of the introduced CEBm4 and cell nucleus, respectively.

Additional References:

1. Zheng, K.W., J.Y. Zhang, Y.D. He, J.Y. Gong, C.J. Wen, J.N. Chen, Y.H. Hao, Y. Zhao, and Z. Tan (2020) Detection of genomic G-quadruplexes in living cells using a small artificial protein. *Nucleic Acids Res*, **48**, 11706-11720.
2. Amrane, S., M. Adrian, B. Heddi, A. Serero, A. Nicolas, J.-L. Mergny, and A.T. Phan, (2012) Formation of Pearl-Necklace Monomorphic G-Quadruplexes in the Human CEB25 Minisatellite. *J Am Chem Soc*, **134**, 5807-5816.
3. Markham, N.R. and M. Zuker, *UNAFold*, in *Bioinformatics: Structure, Function and Applications*, J.M. Keith, Editor. 2008, Humana Press: Totowa, NJ. p. 3-31.
